# Schroth exercises added to bracing for adolescent idiopathic scoliosis: a systematic review and meta-analysis

**DOI:** 10.3389/fspor.2026.1919698

**Published:** 2026-09-23

**Authors:** Kun Yang, Qiuyu Cen, Jiayu Li, Anren Zhang

**Affiliations:** 1 School of Health Preservation and Rehabilitation, Chengdu University of Traditional Chinese Medicine, Chengdu, China; 2 Department of Rehabilitation Medicine, Shanghai Fourth People’s Hospital Affiliated to Tongji University School of Medicine, Shanghai, China

**Keywords:** adolescent idiopathic scoliosis, bracing, exercise therapy, GRADE, meta-analysis, Schroth exercises, scoliosis-specific exercise

## Abstract

**Background:**

Bracing is the principal non-operative treatment for skeletally immature adolescents with idiopathic scoliosis. Schroth exercises are often adjunctively prescribed; however, their incremental value over bracing alone remains uncertain.

**Objective:**

To evaluate the effectiveness, safety, adherence, and treatment burden of Schroth or explicitly Schroth-based exercises added to bracing compared with bracing alone.

**Methods:**

This PROSPERO-registered systematic review and meta-analysis followed the Preferred Reporting Items for Systematic Reviews and Meta-Analyses (PRISMA) 2020 principles. Comparative studies of braced adolescents with idiopathic scoliosis were included. The risk of bias was assessed using version 2 of the Cochrane risk-of-bias tool for randomized trials (RoB 2) or risk of bias in non-randomized studies of interventions (ROBINS-I), and certainty was assessed using Grading of Recommendation Assessment, Development and Evaluation (GRADE). Primary continuous synthesis used postintervention/final values within prespecified clinically comparable follow-up and brace-dose contexts; change score, imputed standard deviation, mixed follow-up, abstract-limited, unequal brace dose, and non-randomized analyses were treated as sensitivity and exploratory evidence.

**Results:**

Fourteen studies were included, comprising nine randomized and five non-randomized comparative studies. Randomized progression evidence was sparse and inconclusive [risk ratio: 0.44, 95% confidence interval (CI): 0.07–2.82; 71 participants]. Primary endpoint/final-value continuous analyses favored combined treatment for the Cobb angle [mean difference (MD): 3.78°, 95% CI: 2.27–5.29; 330 participants] and angle of trunk rotation (MD: 1.82°, 95% CI: 0.16–3.48; 220 participants), but heterogeneity was substantial and certainty was very low. Harm, adherence, and burden were reported incompletely.

**Conclusions:**

Schroth or Schroth-based exercises added to bracing may improve the short-term Cobb angle, while trunk-rotation effects are directionally favorable but uncertain. The evidence was insufficient to determine additional effects on the quality of life, pain, respiratory function, posture, chest-wall or rib-cage mobility, pelvic alignment, balance, gait, long-term progression, surgery, adherence, harm, or treatment burden. Schroth exercises should be framed as adjuncts to prescribed bracing, not as replacements for adequate brace wear or reasons to reduce the brace dose. Future trials should evaluate radiographic, postural, respiratory, functional, adherence, safety, burden, and participation outcomes.

**Systematic Review Registration:**

https://www.crd.york.ac.uk/PROSPERO/view/CRD420261397961, PROSPERO CRD420261397961.

## Introduction

Adolescent idiopathic scoliosis (AIS) is a three-dimensional spinal deformity that may progress during a pubertal growth spurt (1). Progressive curves can result to visible trunk asymmetry, body-image concerns, impaired health-related quality of life, pain in some patients, and the potential need for spinal fusion. Clinical decisions, therefore, depend not only on the Cobb angle at presentation but also on skeletal maturity, remaining growth, curve pattern, prior progression, brace response, and adherence capacity.

Brace treatment is the main conservative intervention for skeletally immature adolescents at risk of progression. Its therapeutic rationale is primarily mechanical: a brace attempts to reduce asymmetric loading and restrain progression while growth remains (1, 2). Brace effectiveness is closely linked to the brace design, in-brace correction, prescribed wear time, and real-world adherence (2, 7–9). The same features that make bracing effective can also shape the daily movement environment of adolescents. Long wear schedules may alter comfort during school, sleep, activity, and social participation and create heat, skin pressure, appearance, and psychosocial burdens (8, 11). Bracing and AIS may also intersect with trunk movement, balance, and gait adaptation (46–48), while the broader AIS rehabilitation literature has emphasized respiratory and thoracic functions as relevant outcomes rather than purely radiographic endpoints (1, 15, 16).

General AIS natural history, progression risk, brace-study criteria, skeletal maturity assessment, brace-dose response, surgical rate context, and bracing burden have been described in prior clinical and review literature (3–11). These background issues are important because the value of adjunctive exercise depends on the progression risk that bracing is intended to manage, as well as the real-world feasibility of adding another rehabilitation task.

For clinicians and researchers focused on non-surgical AIS management, this trade-off is important. A brace is not just a radiographic intervention; it is also a device worn during growth, school, sleep, physical activity, and social participation. A clinically useful adjunct should therefore be judged not only by whether it changes the Cobb angle but also by whether it helps adolescents maintain active postural control, respiratory mechanics, trunk and pelvic coordination, comfort, confidence, and sustainable participation while preserving adequate brace wear.

Schroth exercises are a scoliosis-specific exercise approach that trains active three-dimensional self-correction, axial elongation, postural derotation, rotational angular breathing, stabilization of corrected alignment, and the transfer of corrected posture into daily activities (1, 12, 13, 39). These mechanisms are clinically plausible as adjuncts to bracing because trials and secondary analyses of Schroth or scoliosis-specific exercise have reported effects or outcome assessment in active correction, quality of life, muscle endurance, pulmonary function, exercise tolerance, surface topography, and pelvic or spinal alignment (12–16, 52, 53). These findings support a broader rehabilitation rationale involving active correction outside the brace, trunk symmetry, rib-cage and thoracic expansion strategies, respiratory awareness, neuromuscular control, and patient engagement. However, Schroth should not be assumed to replace the sustained external corrective forces of appropriately prescribed braces.

Previous reviews have often combined different exercise schools, mixed braced and non-braced populations, or pooled heterogeneous comparators. A recent network meta-analysis ranked Schroth plus bracing highly among conservative options (14); however, the practical question remains narrower: whether Schroth adds benefit for adolescents already prescribed a brace. Recent reviews in *Frontiers in Sports and Active Living* have also synthesized AIS exercise evidence for pulmonary/exercise tolerance outcomes and scoliosis-specific exercise protocol ranking; however, these reviews did not resolve the specific add-on-to-bracing comparison addressed here (15, 16).

For readers interested in exercise and rehabilitation, this review focuses on the question of additive rehabilitation rather than the broader issue of whether scoliosis-specific exercise is useful on its own or how different exercise schools rank against one another. The synthesis, therefore, prioritizes pure add-on comparisons, comparable brace-dose contexts, and outcomes that are significant when Schroth training is used to support, rather than replace, prescribed bracing.

## Methods

### Registration and reporting

This systematic review was registered in PROSPERO (CRD420261397961) and followed the Preferred Reporting Items for Systematic Reviews and Meta-Analyses (PRISMA) 2020 principles (17). Eligibility criteria and outcomes followed the protocol. Search reporting followed the PRISMA-S principles (18), and protocol deviations are documented in Supplementary Data Sheet 1. The Cochrane Handbook (19) guided data handling and synthesis , and reporting was checked against sports, exercise, and musculoskeletal rehabilitation guidance where applicable (20).

### Eligibility criteria

The eligibility criteria were structured using the population, interventions, comparators, outcomes, and study designs (PICOS) framework. The population was braced adolescents with idiopathic scoliosis; the intervention was Schroth or explicitly Schroth-based scoliosis-specific exercise added to bracing; the comparator was bracing alone; outcomes included the Cobb angle, curve progression, treatment failure or surgery, angle of trunk rotation (ATR), quality of life, pain, respiratory outcomes, adherence, adverse events, and burden; and eligible study designs were randomized, quasi-randomized, and comparative non-randomized studies.

Eligible participants were adolescents, approximately 10–18 years old, with idiopathic scoliosis, who were receiving brace treatment. Studies with mixed age ranges were eligible only when adolescent idiopathic scoliosis data were extractable or when most participants were adolescents with AIS. Congenital, neuromuscular, syndromic, degenerative, traumatic, postsurgical, and adult scoliosis populations were excluded unless the AIS adolescent data could be separated.

The intervention was Schroth exercises or explicitly Schroth-based scoliosis-specific exercises delivered in addition to the bracing. Programs can be supervised, home-based, or a combination of both. The comparator was brace treatment alone, without Schroth exercises or any other structured scoliosis-specific exercise program. Randomized controlled trials, quasi-randomized studies, and comparative non-randomized studies were eligible. Case reports, case series, single-arm studies, cross-sectional studies, reviews, editorials, protocols, and conference abstracts without sufficient outcome data were excluded.

### Search and study selection

We searched PubMed/MEDLINE, Embase, CENTRAL, CINAHL Complete, Web of Science Core Collection, PEDro, ClinicalTrials.gov, WHO ICTRP, CNKI, Wanfang Data, and VIP Chinese Journal Database from inception to the final search date without language or date restrictions. Reference-list and forward-citation checks were also performed. Full source-level strategies, counts, and custody materials are provided in Supplementary Data Sheet 1.

No submitted manuscripts, personal communications, author-supplied unpublished outcome data, or other private original data were included. Records must be publicly retrievable from journals, bibliographic databases, registries, institutional repositories, or other documented public sources. Publicly available theses and abstract-limited reports were handled transparently in the evidence map and sensitivity or exploratory analyses, where applicable, and they did not determine the primary endpoint/final-value synthesis.

### Data extraction and risk of bias

Two reviewers extracted the study characteristics, participant features, brace type and prescription, Schroth program content and dose, comparator details, follow-up time points, outcomes, brace adherence, exercise adherence, withdrawals, adverse events, and treatment-burden information. Outcomes included Cobb angle change, progression, ATR, quality of life, pain, respiratory outcomes, patient-reported outcomes, and, when reported, posture or surface-topography measures, thoracic or rib-cage mobility, pelvic alignment, balance, gait, or other whole-chain functional measures. Disagreements were resolved through discussion. Randomized studies were assessed using version 2 of the Cochrane risk-of-bias tool for randomized trials (RoB 2) and non-randomized studies were assessed using risk of bias in non-randomized studies of interventions (ROBINS-I) (19, 21).

### Outcomes and synthesis

The primary outcomes were Cobb angle change, curve progression, and treatment failure or surgery. Secondary outcomes included ATR, quality of life, pain, respiratory outcomes, brace adherence, exercise adherence, adverse events, and patient-reported outcomes. Postural, thoracic, rib-cage, pelvic, balance, gait, and activity-participation outcomes were handled narratively when available because they were not reported consistently enough for pooled add-on-to-bracing estimates. Continuous outcomes were analyzed as mean differences (MDs), with positive values indicating greater improvement favoring Schroth plus bracing. Dichotomous outcomes were analyzed as risk ratios (RRs), with values <1 favoring the combined treatment for progression.

Randomized and non-randomized evidence was synthesized separately. Primary continuous synthesis used postintervention/final values within prespecified clinically comparable follow-up and brace-dose contexts, where available. Primary endpoint/final-value meta-analyses used inverse-variance DerSimonian–Laird random-effects models (22), and Hartung–Knapp intervals were reported as small-sample uncertainty checks. Change score, imputed standard deviation (SD), mixed follow-up, abstract-limited, unequal brace dose, and non-randomized analyses were treated as sensitivity or exploratory evidence. MDs with 95% confidence intervals (CIs) were calculated, with positive values favoring combined treatment for Cobb angle reduction, ATR reduction, or 22-item Scoliosis Research Society questionnaire (SRS-22) improvement. Statistical calculations were performed using Python 3.12.13 with a standard-library analysis script (csv and math modules; no external statistical package) that implemented the inverse-variance, DerSimonian–Laird, REML, Hartung–Knapp, prediction-interval, and leave-one-out procedures reported in the Supplementary Data Sheet 1. The analysis dataset, submitted sensitivity table, Python reproduction script, and independent R/metafor replication script are provided in Supplementary Data Sheet 1.

Where change SDs, final SDs, or event counts were unavailable, missing data were listed in an author-contact log and request template. Change SDs were estimated only in sensitivity analyses using prespecified within-person correlations (*r* = 0.25, 0.50, 0.75), with *r* = 0.50 as the central scenario (19). Studies lacking extractable SDs, event counts, or comparable follow-up were retained narratively or in relevant exploratory strata.

Small-study effects and possible publication bias were explored using funnel plots and Egger’s test only when at least 10 studies were available for an outcome-specific meta-analysis. When fewer studies were available, publication bias was qualitatively assessed within the Grading of Recommendation Assessment, Development and Evaluation (GRADE) assessment.

Scopus and SinoMed/CBM were prespecified but could not be searched because institutional access was unavailable, and CENTRAL was only partially captured because full export/authentication failed. These deviations were not considered completed searches. Access-attempt logs, rerun strategies, CENTRAL partial-capture notes, raw-export custody materials, and PRESS-style search self-audit materials are provided in Supplementary Data Sheet 1.

### Certainty of evidence

The certainty of evidence for each critical and important outcome was assessed using GRADE, considering the risk of bias, inconsistency, indirectness, imprecision, and publication bias (23).

## Results

### Study selection

A total of 1,911 records were imported from all sources and 908 unique records remained after deduplication (Figure 1). All unique records were screened based on their titles and abstracts. Forty-three full texts were assessed, 29 were excluded for various reasons, and 14 studies were included. The excluded full texts, screening decisions, and reasons are provided in Supplementary Data Sheet 1. Supplementary Data Sheet 1 also presents the deduplication summaries and duplicate-removal outputs behind the PRISMA identification count.

**Figure 1:**
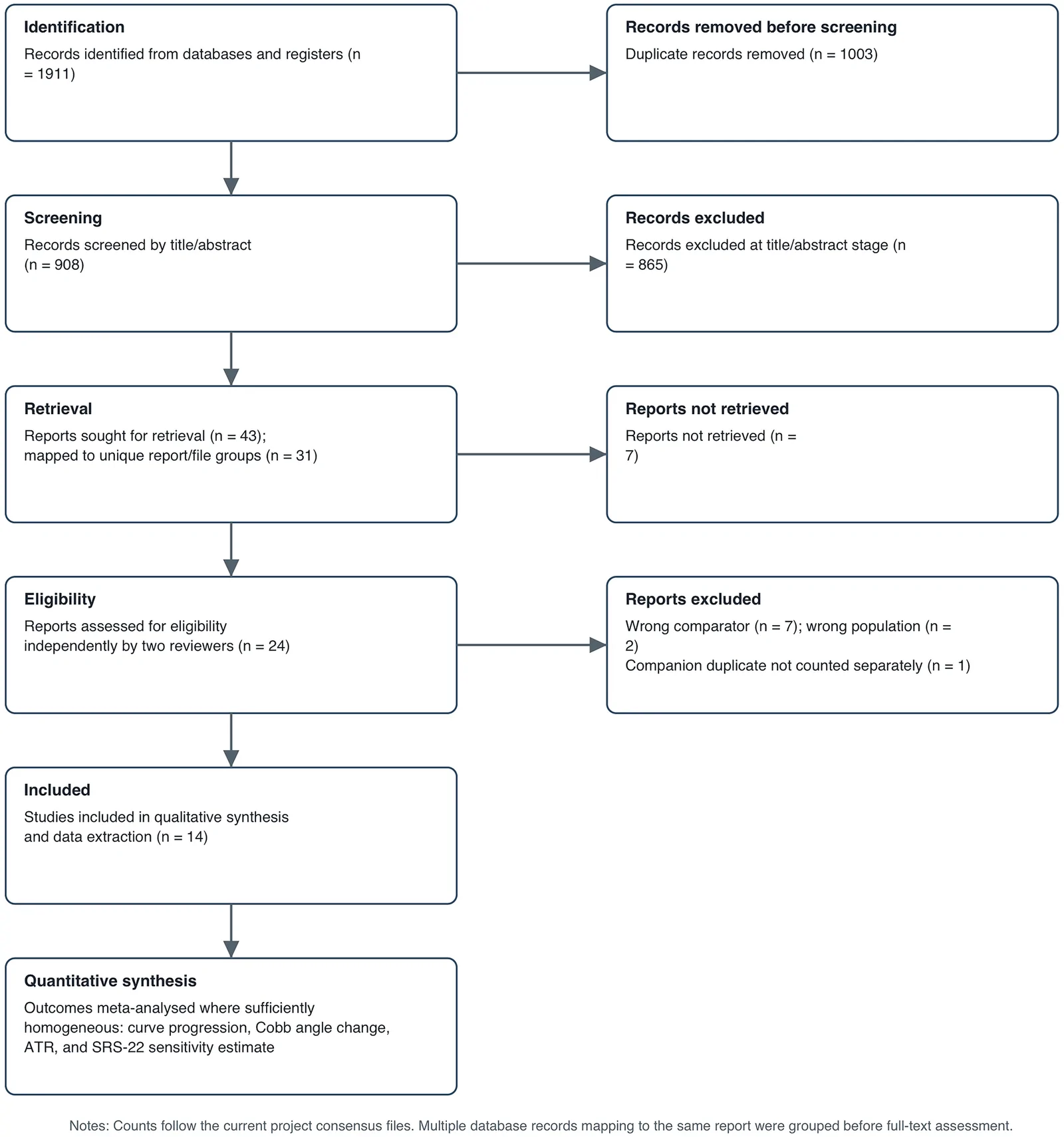
PRISMA 2020 flow diagram. Flow of records and reports through identification, screening, retrieval, eligibility assessment, and inclusion. The counts reflect the two-reviewer full-text consensus and grouped report-level assessments.

### Study characteristics

The 14 included studies evaluated Schroth or explicitly Schroth-based exercises as an adjunct to bracing in adolescents with idiopathic scoliosis (Table 1) (24–37). Nine studies were randomized comparative studies, and five were non-randomized comparative studies. Brace approaches included thoracolumbosacral orthoses, Cheneau-type braces, Providence nighttime bracing, Gensingen/Cheneau principle braces, and three-dimensional printed braces. The exercise programs varied in terms of supervision, frequency, duration, home practice, and therapist training. Several studies contributed only to narrative synthesis or sensitivity analysis because of missing SDs, incomplete tabular reporting, or clinical indirectness.

**Table 1:** Characteristics of included studies.

Study	Country/design	Participants	Baseline AIS profile	Brace/comparator	Schroth adjunct	Follow-up/outcomes	RoB	Analysis role
Shi (24) (FTS-001)	China; reported randomized comparative trial	51/50 analyzed	Age 13.85 vs. 14.09 years; Risser 0-IV; Cobb 30.74 (3.86) vs. 31.32 (3.54) deg.	Symmetric thoracolumbosacral orthosis; 2-week adaptation, then ≥22 h/day for 22 weeks; comparator orthosis alone	Clinical rehabilitation Schroth training; 3 sessions/week, 1.5 h/session, 24 weeks	12 and 24 weeks; Cobb, ATR, progression	RoB 2: Some concerns	Narrative; reported randomized trial, change SDs unavailable
Liang et al. (25) (FTS-002)	China; reported randomized comparative trial	55/55 analyzed	Age 10–18 years; Risser 0–4; Cobb 33.42 (4.25) vs. 33.25 (4.47) deg.	3D-printed spinal orthosis for 6 months; >18 h/day after 2-week adaptation; comparator 3D printed orthosis plus conventional rehabilitation/education	Therapist-guided Schroth gymnastics; 3 sessions/week, 90 min/session, 6 months	6 months; Cobb, ATR, progression	RoB 2: Some concerns	Narrative synthesis; Cobb values extractable, but the change SDs were not directly reported
Liu et al. (26) (FTS-003)	China; reported randomized comparative trial	19/20 analyzed	Age 12.47 vs. 12.80 years; Risser 0–4; Cobb 33.38 (5.27) vs. 34.19 (6.57) deg.	Modified Cheneau full-time rigid brace; ≥20 h/day for 24 weeks; comparator same brace with education/follow-up	Weekly hospital guidance plus home training; 5 sessions/week, 60 min/session, 24 weeks	24 weeks; Cobb, ATR, progression	RoB 2: Some concerns	Reported randomized trial event data for progression; narrative for change outcomes without SD
Kwan et al. (27) (FTS-007)	Hong Kong, China; prospective non-randomized	24/24 analyzed	Age 12.3 vs. 11.83 years; Risser 0–2 or radius/ulna stage criterion; Cobb 25–40 deg required, group means not extractable	Rigid underarm orthosis; minimum 18 h/day; comparator 1:1 historical matched bracing-only cohort	8-week outpatient Schroth program with private sessions every 2 weeks plus home practice	Mean 18.1 vs. 38.75 months; Cobb, ATR, progression, SRS-22	ROBINS-I: Serious	Supportive non-randomized progression evidence only
Lee et al. (28) (FTS-008)	Republic of Korea; reported randomized comparative trial	14/14 analyzed	Age 10.71 vs. 12.07 years; Risser 0–2; Cobb 44.72 (15.38) vs. 39.23 (6.37) deg.	Gensingen/Cheneau-principle brace; at least 18 h/day for 12 weeks; comparator brace only	Supervised Schroth sessions; 3 sessions/week, 50 min/session, 12 weeks	Baseline, 6 weeks, and 12 weeks; Cobb, ATR, progression	RoB 2: Some concerns	Change-score/no-imputation sensitivity evidence; raw final SDs not available for the primary endpoint/final-value synthesis
Zapata et al. (29) (FTS-009)	USA; prospective non-randomized	37/37 analyzed	Age 12.7 vs. 12.1 years; Risser 0 required; Cobb 24 (13) vs. 25 (13) deg.	Providence nighttime brace; comparator standard nighttime bracing without PSSE	One-on-one outpatient PT within 6 months plus home exercise at least 5 days/week, minimum 15 min/day, for at least 1 year	1 year and final visit at skeletal maturity or surgery; Cobb, progression	ROBINS-I: Serious	Supportive non-randomized progression/Cobb evidence
Kastrinis et al. (30) (FTS-010)	Greece; retrospective/historical non-randomized	25/25 analyzed	Age 12.7 vs. 13.5 years; Risser 0–4; Cobb 31.7 (9.4) vs. 33.2 (11.1) deg.	3D asymmetric Schroth-compatible CAD/CAM brace; 18–22 h/day; comparator brace alone	Certified ISST Schroth therapists; twice/week 1 h plus home exercise 30 min; 6 months	Initial in-brace at 1 month and follow-up in-brace radiographs; Cobb, progression	ROBINS-I: Serious	Secondary/narrative; mainly in-brace correction
Fang et al. (31) (FTS-012)	China; retrospective/historical non-randomized	54/138 analyzed	Age 12.0 vs. 12.29 years; Risser 0–2; Cobb 31.89 (4.50) vs. 32.04 (5.42) deg.	Cheneau-type thoracolumbosacral orthosis; ≥20 h/day until treatment endpoint/weaning; comparator Cheneau brace alone	Physiotherapist-supervised SBP plus daily home exercises; 3 days/week for 6 weeks, followed by a home program	Pretreatment, first in-brace, and end of treatment; Cobb, progression	ROBINS-I: Serious	Supportive non-randomized Cobb/progression evidence
Wu (32) (FTS-014)	China; reported randomized comparative trial	38/38 analyzed	Abstract-limited; age, skeletal maturity, and baseline Cobb values not extractable from the available abstracts	3D-printed spinal orthosis; comparator 3D printed spinal orthosis alone; additional brace details abstract-limited	Schroth orthopedic gymnastics; delivery details abstract-limited; reported treatment duration 6 months	6 months; Cobb, ATR, progression	RoB 2: High	Narrative and high-sensitivity evidence only; incompletely extractable
Wu et al. (33) (FTS-015)	China; reported randomized comparative trial	60/60 analyzed	Baseline comparability reported; exact baseline values not reported in the available abstracts	Brace orthosis; exact brace type and prescribed wear schedule not reported in the extractable abstract; comparator brace alone	Schroth orthopedic gymnastics; delivery details not reported in the extractable abstract	Postintervention time point not reported in the available abstract; Cobb, ATR, progression	RoB 2: High	Abstract-limited high-risk exploratory imputed-SD sensitivity stratum for Cobb and ATR only
Yao (34) (FTS-016)	China; reported randomized comparative trial	16/16 analyzed	Age 14.31 vs. 14.38 years; Risser 2–4; Cobb 30.63 (5.56) vs. 29.59 (6.78) deg.	3D printed Gensingen (GBW) brace; after 2-week adaptation, ≥18 h/day until follow-up; comparator GBW brace alone	Modified Schroth PSSE; 4 weeks supervised, then home/continued therapist training; 12-week formal program, then maintenance	Mean follow-up 16.59 vs. 15.00 months; Cobb, ATR, progression	RoB 2: High	Change-score/no-imputation sensitivity evidence; high RoB due to attrition; not in the primary endpoint/final-value synthesis
Kong (35) (FTS-017)	China; non-randomized comparative thesis	58/61 analyzed	Age 10–17 years; Risser ≤3; baseline Cobb values not reported in extractable tables	Cheneau thoracolumbosacral brace; worn except during sleeping and bathing; comparator Cheneau brace alone; Schroth-only arm excluded	Certified Schroth therapist; intensive training cycles plus home practice; at least 12 months with 3-month review intervals	6 and 12 months; Cobb, ATR, progression	ROBINS-I: Serious	Narrative and high-sensitivity evidence only; non-randomized thesis with incomplete extractable variance data
Kyrkousis et al. (36) (FTS-019)	Greece; reported randomized comparative trial	40/40 analyzed	Age 13.67 vs. 13.55 years; Risser 0–3; Cobb 33.43 (5.98) vs. 34.65 (3.08) deg.	Scoliosis brace, type not specified; reported mean daily wear about 18 h/day; comparator brace plus physical activity advice	Clinic-based Schroth supervised by trained physiotherapist; 3 sessions/week, 60 min/session, 12 months, with a 6-month postintervention follow-up	Baseline, 12 months, and 18 months; Cobb, ATR, progression, SRS-22	RoB 2: Some concerns	Included in the primary endpoint/final-value Cobb/ATR synthesis; SRS-22 and change-score analyses remain sensitivity evidence
Meng et al. (37) (FTS-021)	China; reported randomized comparative trial	25/22 analyzed	Age 13.46 vs. 13.75 years; Risser 0–III; Cobb 34.16 (10.28) vs. 31.63 (8.66) deg.	Cheneau brace; intervention 14–18 h/day after adaptation; control full-time brace 20–24 h/day	Certified Schroth PT; 4–5 initial outpatient sessions, then weekly sessions; 40–60 min/session; 6-month treatment	Baseline, 6 months, and 12 months for Cobb; 6 months for ATR/SRS-22; progression	RoB 2: Some concerns	Pragmatic indirect no-imputation sensitivity only because the brace dose differs by design

Schroth or explicitly Schroth-based exercises plus bracing compared with bracing alone in adolescent idiopathic scoliosis.

AIS, adolescent idiopathic scoliosis; ATR, angle of trunk rotation; PSSE, physiotherapeutic scoliosis-specific exercises; RoB, risk of bias; SBP, Schroth best practice; SRS-22, 22-item Scoliosis Research Society questionnaire. The analysis role reflects the current synthesis plan.

### Risk of bias

No reported randomized study was judged to have a low risk of bias. Six were judged to have some concerns and three had a high risk of bias. Common limitations included unclear allocation concealment, incomplete assessor masking, limited protocol availability, uncertain selective reporting, incomplete adherence measurement, and postrandomization exclusion. All five non-randomized studies were judged to have a serious risk of bias, mainly because of confounding, treatment-choice allocation, historical controls, attrition, and completer-only selection (Figure 2).

**Figure 2:**
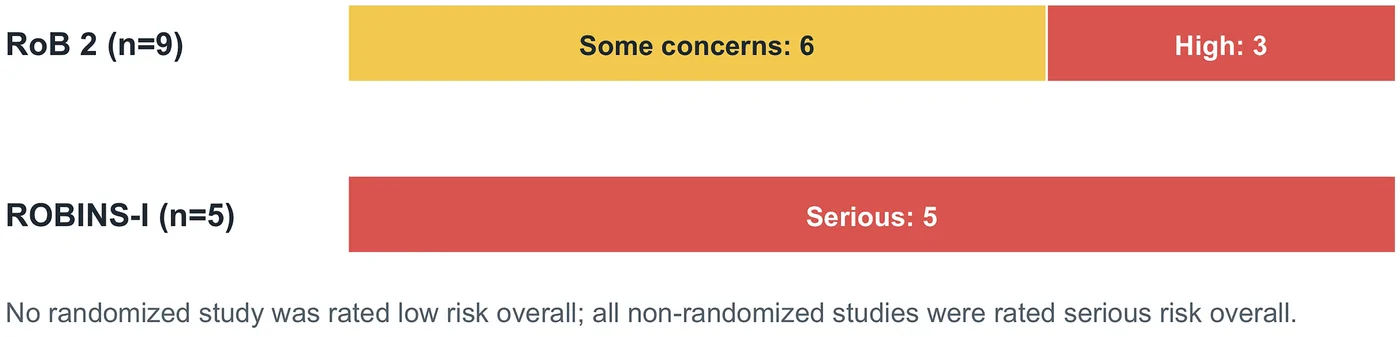
Risk-of-bias summary. Overall risk-of-bias judgments for studies reported as randomized comparative studies assessed using RoB 2 and non-randomized studies assessed using ROBINS-I. Detailed traffic-light plots are provided in Supplementary Data Sheet 1.

### Curve progression and treatment failure

Randomized evidence for curve progression was limited to two small studies and was statistically imprecise (RR 0.44, 95% CI 0.07–2.82; 71 participants; Figure 3). The point estimate favored the combined treatment, but the confidence interval was compatible with a substantial benefit and no reliable difference. Non-randomized studies showed a more favorable direction, but they were not pooled with randomized evidence because all were at serious risk of bias and vulnerable to confounding factors such as motivation, adherence, treatment access, and baseline prognosis.

**Figure 3:**
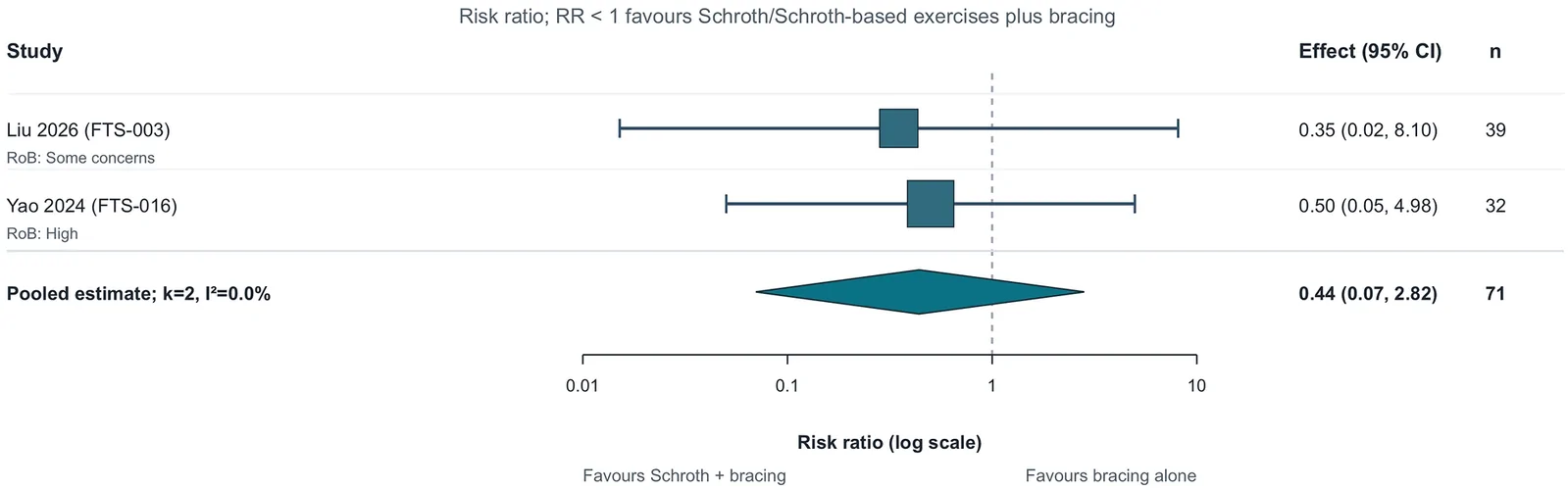
Curve progression/treatment failure. Randomized evidence comparing Schroth or Schroth-based exercises plus bracing with bracing alone. Risk ratios less than 1 favor the combined treatment.

Treatment failure, surgery, and escalation of care could not be meaningfully pooled. Definitions varied, direct event counts were sparse, and most studies did not follow participants until skeletal maturity or brace weaning. Therefore, the current evidence cannot determine whether adding Schroth reduces the risk of reaching a surgical threshold or requiring operative treatment.

### Cobb angle

In the primary endpoint/final-value continuous synthesis, four randomized pure add-on studies provided raw final Cobb angle means and SDs within clinically comparable brace-dose contexts (330 participants; Figure 4A). The pooled MD was 3.8° favoring combined treatment (95% CI 2.3–5.3; *I*^2^ = 69.9%; Hartung–Knapp CI 1.8–5.7). Change-score strata were retained as sensitivity evidence because directly calculable change SDs were sparse.

**Figure 4:**
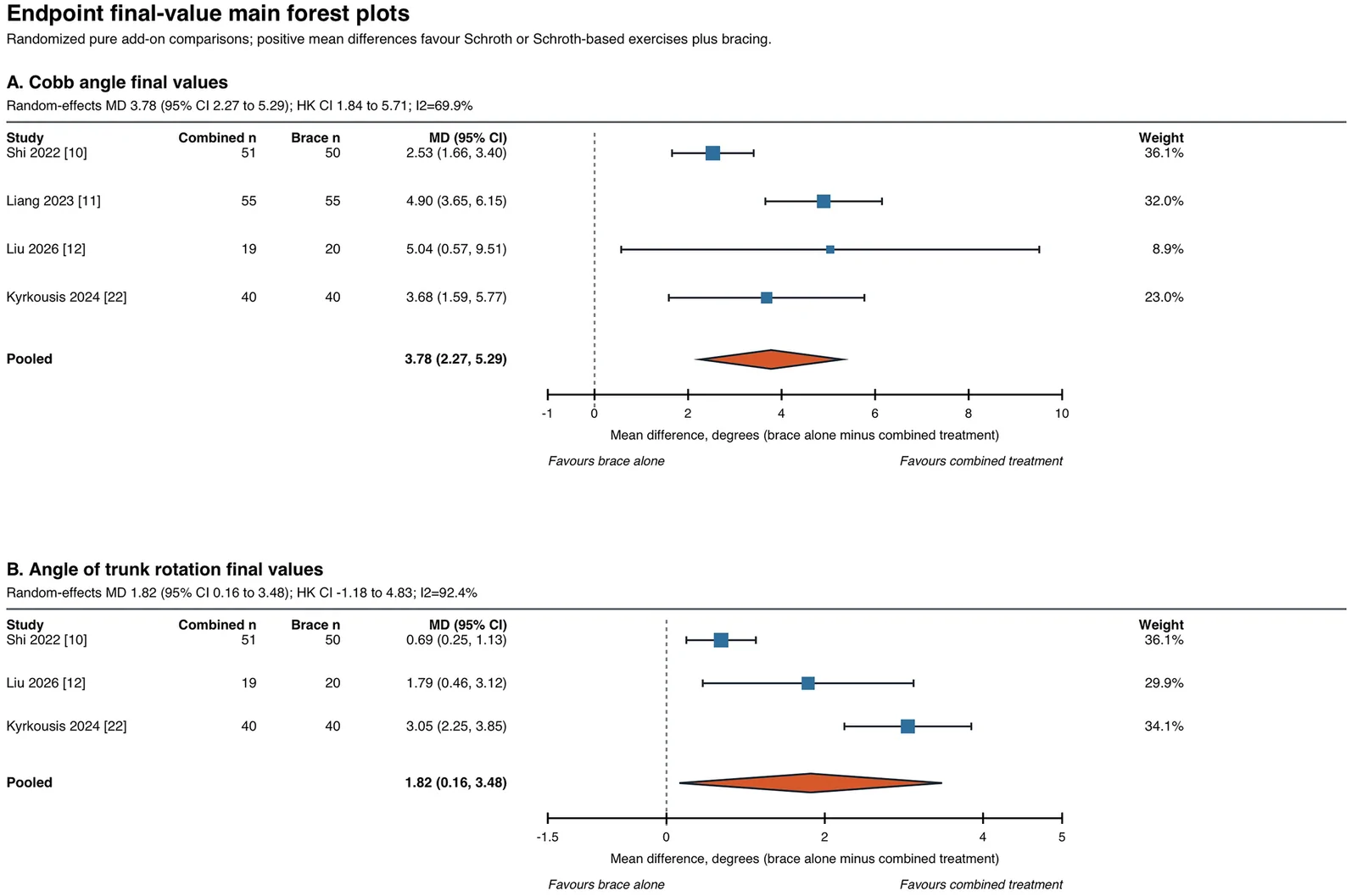
Endpoint Cobb angle and angle of trunk rotation, main forest plots. **(A)** Postintervention raw final Cobb angle values. **(B)** Postintervention raw final ATR values. Mean differences are brace-alone minus combined-treatment final values, so estimates greater than 0 favor Schroth or Schroth-based exercises plus bracing. Pooled estimates use DerSimonian–Laird random effects; Hartung–Knapp intervals are reported in the panel text and manuscript.

Sensitivity analyses using imputed change SDs, mixed follow-up, abstract-limited evidence, or unequal brace-dose contexts allowed otherwise relevant studies to be examined. These analyses were directionally supportive but less direct and more assumption-dependent; therefore, they were used to test robustness rather than to replace the primary endpoint/final-value continuous synthesis. Meng et al. was classified *a priori* as pragmatic indirect unequal-brace-dose evidence because the intervention group received Schroth support with a 14–18 h/day brace prescription, whereas the comparator received 20–24 h/day bracing. Therefore, we addressed whether an exercise-supported lower-brace-dose strategy can perform comparably to full-time bracing, not the pure add-on effect of Schroth when the brace dose is otherwise comparable.

For key sensitivity outcomes, conservative uncertainty estimation did not fully support a stable conclusion: Hartung–Knapp intervals were wider, some crossed no effect, and prediction intervals were wide, where estimable. These analyses provide sensitivity evidence, not replacements for the primary endpoint/final-value synthesis.

### Angle of trunk rotation

For ATR, the primary endpoint/final-value continuous synthesis included three randomized pure add-on studies (220 participants; Figure 4B) and favored combined treatment (MD 1.8°, 95% CI 0.2–3.5; *I*^2^ = 92.4%; Hartung–Knapp CI −1.2 to 4.8). The larger heterogeneity and the wide small-sample interval mean that the direction is supportive but uncertain. Change scores, imputed SDs, and pragmatic unequal brace-dose analyses were retained as sensitivity or exploratory evidence.

### Quality of life and adverse events

Only FTS-019 provided a directly alignable SRS-22 total-score estimate after change-SD imputation (MD 0.8, 95% CI 0.5–1.1 at *r* = 0.50). Pain, respiratory function, thoracic or rib-cage mobility, detailed posture or surface-topography measures, pelvic alignment, balance, gait, and activity-participation outcomes were absent, inconsistently reported, or not extractable for a pooled add-on-to-bracing analysis. Brace adherence, exercise dose, withdrawals, adverse events, and treatment burden were extracted into a dedicated table because these variables affect clinical interpretation, feasibility, and external validity, but were inconsistently reported (Table 2).

**Table 2:** Brace adherence, exercise dose, withdrawals, adverse events, and treatment burden/source of missing data.

Study	Brace adherence	Exercise dose/adherence	Withdrawals/missing data	Adverse events	Burden/source of missing data
Shi (24)	Prescribed ≥22 h/day after 2-week adaptation; clinical follow-up; no objective sensor; actual hours NR	Schroth 3 sessions/week, 1.5 h/session, 24 weeks; home exercise unclear; exercise adherence NR	0/51 vs. 0/50 withdrawals reported	NR	High prescribed brace time plus supervised exercise; missing adherence and harm data from non-reporting
Liang et al. (25)	Prescribed >18 h/day from week 3 for 6 months; no objective monitoring; actual hours NR	Schroth 3 sessions/week, 90 min/session, 6 months; adherence NR	Withdrawals NR	NR	Substantial time commitment; missing data mainly from incomplete reporting
Liu et al. (26)	Prescribed ≥20 h/day for 24 weeks; weekly phone/parent/therapist follow-up; no sensor; actual hours NR	Schroth 5 sessions/week, 60 min/session, 24 weeks; home training on non-hospital days; quantitative adherence NR	0/19 vs. 0/20 withdrawals reported	NR	Frequent supervised/home exercise; harm and quantitative adherence missing from non-reporting
Kwan et al. (27)	Minimum 18 h/day; clinical compliance rating; good compliance 70.8% vs. 79.2%; no objective sensor	Private sessions every 2 weeks for 8 weeks, plus home exercise ≥5 days/week; caregiver verification and therapist checklist	Withdrawals NR	No adverse effects observed	Home program burden documented indirectly; withdrawal data missing
Lee et al. (28)	At least 18 h/day for 12 weeks; adherence not objectively monitored; actual hours NR	Schroth 3 sessions/week, 50 min/session, 12 weeks; adherence not objectively monitored	0/21 vs. 0/21; all participants analyzed	Potential risks discussed, but separate adverse-event counts NR	Short but intensive program; harm data limited by non-reporting of counts
Zapata et al. (29)	Nighttime Providence brace; temperature sensor every 15 min; course wear 8.0 ± 2.9 vs. 7.3 ± 2.7 h/night	Home PSSE ≥15 min/day, 5 days/week for ≥1 year plus ≥8 h outpatient PT; adherence 64% at 6 months and 51% at 1 year	21/58 vs. 20/57 withdrew or were lost to follow-up	NR	Long home program; attrition and declining adherence are major external-validity concerns
Kastrinis et al. (30)	Full-time 18–22 h/day; self-reported compliance 86.3% vs. 81.3% at 6 months	Supervised twice/week, 1 h/session plus home 4 times/week, 30 min/session for 6 months; exercise adherence not separately quantified	Withdrawals NR	NR	Combined supervised and home schedules; self-reported adherence and missing withdrawals/harm counts
Fang et al. (31)	Prescribed ≥20 h/day; logbooks with parent/therapist verification; actual 21.58 ± 0.66 vs. 20.92 ± 0.79 h/day	Supervised 3 days/week for 6 weeks plus daily 30–45 min home exercise; logbook adherence	Lost-to-follow-up participants excluded; analyzed cohort only	Formal adverse-event counts NR; brace-related pain/HRQoL concerns discussed	High brace dose and daily exercise; selection/missing data from excluding incomplete follow-up
Wu (32)	NR in the extractable abstract	NR in the extractable abstract	NR	NR	Abstract-limited reporting
Wu et al. (33)	NR in the extractable abstract	NR in the extractable abstract	NR	NR	Abstract-limited reporting
Yao (34)	After 2-week adaptation, ≥18 h/day; eligibility/exclusion based on wear ≥18 h/day; no objective sensor; actual hours NR	Weeks 1–4: 2–3 sessions/week, 45–60 min; thereafter 2–3/week, 30–45 min with WeChat/therapist follow-up	4/20 vs. 4/20 excluded as outliers/lost/ineligible for final analysis	No formal adverse-event table; soreness after the Schroth training mentioned	Exclusions after allocation and non-objective adherence monitoring limit the interpretability
Kong (35)	Brace worn except during sleeping/bathing; brace maker supervised daily wearing, weekly feedback; actual hours NR	Intensive 5–7 sessions/week, 4–6 h/day for 2–7 weeks, then ≥1 h/day daily-life training; quantitative adherence NR	Withdrawals NR; inclusion required ≥1 year treatment/training completion	Treatment risk described as low; no adverse-event counts	Very high exercise burden; completion-based inclusion may select adherent participants
Kyrkousis et al. (36)	Mean daily brace wear 18.6 ± 1.97 vs. 17.95 ± 1.88 h/day; measurement not clearly reported	Schroth 3 sessions/week, 60 min/session for 12 months; missed sessions rescheduled; no participant missed >7 days	ITT retained 40/group; 2 vs. 1 missed 12-month measurement, plus 2 vs. 2 stopped communication by 18 months	No complications or adverse symptoms reported	Long supervised program; follow-up communication loss after the intervention
Meng et al. (37)	Unequal brace dose: 14–18 h/day plus Schroth vs. 20–24 h/day control; actual 14.72 ± 0.85 vs. 21.22 ± 1.15 h/day	4–5 weekly sessions initially; mean exercise 4.9 h/week; logs, checklists, videos, weekly outpatient review	1/26 vs. 4/26 withdrew after randomization; final analysis 25 vs. 22	Not reported as adverse-event counts	Pragmatic brace-dose reduction context; not pure add-on evidence; incomplete harm counts

NR, not reported. Values are extracted as reported; absence of adverse-event reporting should not be interpreted as the absence of harm.

### Brace and exercise adherence

Adherence to reporting varied substantially across studies (Table 2). Only Zapata et al. used objective temperature-sensor brace monitoring, recording Providence nighttime brace wear every 15 min and reporting a mean course wear of 8.0 ± 2.9 versus 7.3 ± 2.7 h/night. Other studies relied on prescription statements, clinical follow-up, self-report, logbooks, parent or therapist verification, brace-maker feedback, WeChat/therapist follow-up, and unclear measurement methods. Fang et al. reported logbook-verified wear of 21.58 ± 0.66 versus 20.92 ± 0.79 h/day; Kastrinis et al. reported self-reported compliance; Kyrkousis et al. reported mean daily brace wear but did not clearly specify the measurement method; Meng et al. reported actual hours in an unequal-brace-dose design; and several Chinese or abstract-limited reports provided only prescribed hours or no quantitative adherence data. Exercise adherence was also inconsistently measured: Zapata et al reported declining home-exercise adherence (64% at 6 months and 51% at 1 year), Fang et al. used logbook adherence; Meng et al. used logs, checklists, videos, and weekly review; and Kyrkousis et al. reported rescheduled missed sessions and no absence longer than 7 days. Most of the remaining studies reported exercise prescriptions without objective or quantitative completion data. Consequently, pooled effects may overestimate real-world effectiveness if the analyzed cohorts are more adherent than typical clinical populations.

### Certainty of evidence

The certainty of evidence was very low for all critical and important outcomes (Table 3). Downgrading reflected the risk of bias, sparse data, indirectness from mixed follow-up or brace-dose contexts, imprecision, and poor reporting of progression, adherence, harm, and treatment burden. Publication bias was qualitatively considered because no outcome-specific meta-analysis included at least 10 studies. The certainty judgment, therefore, reflects not only statistical imprecision but also uncertainty about whether trial protocols, adherence support, and cointervention intensity were comparable across studies.

**Table 3:** Summary of the findings.

Outcome	Anticipated absolute or relative effect	Participants and studies	Certainty of evidence (GRADE)	Interpretation
Cobb angle change from baseline	Primary endpoint/final-value synthesis: MD 3.78° favoring Schroth plus bracing (95% CI 2.27 to 5.29; Hartung–Knapp CI 1.84 to 5.71; *I*^2^ = 69.9%). Change-score, mixed-follow-up, and imputed-SD analyses were supportive sensitivity/exploratory evidence but did not replace the endpoint/final-value synthesis	Primary endpoint/final-value synthesis: 330 participants, four randomized pure add-on studies. Sensitivity/exploratory change-score and imputed-SD analyses included smaller or less directly comparable sets	Very low	Schroth plus bracing may improve Cobb angle more than bracing alone, but the certainty is very low, and heterogeneity remains important
Curve progression	RR 0.44 (95% CI 0.07–2.82).	71 participants, two studies	Very low	We are very uncertain whether Schroth plus bracing reduces curve progression
Treatment failure, surgery, or escalation of care	Not pooled because event definitions and direct event counts were sparse or inconsistently reported	Sparse direct evidence	Very low	Evidence is insufficient to determine whether Schroth plus bracing changes treatment failure or surgery risk
Angle of trunk rotation (ATR)	Primary endpoint/final-value synthesis: MD 1.82° favoring Schroth plus bracing (95% CI 0.16–3.48; Hartung–Knapp CI −1.18 to 4.83; *I*^2^ = 92.4%). Change scores, unequal brace dose, and imputed-SD analyses were retained as sensitivity/exploratory evidence	Primary endpoint/final-value synthesis: 220 participants, three randomized pure add-on studies. Sensitivity/exploratory analyses included change scores, unequal brace dose, or imputed-SD evidence	Very low	Effects on trunk rotation are directionally favorable but remain uncertain because heterogeneity is substantial and certainty is very low
Quality of life/SRS-22 total score	FTS-019 single-study imputed-SD sensitivity suggested a possible SRS-22 total-score difference (MD 0.82, 95% CI 0.51–1.13; *r* = 0.50), but patient-reported outcome evidence was sparse and sensitivity-based	80 participants, one study	Very low	Effects on SRS-22 total score and other patient-reported outcomes remain uncertain because the quantitative estimate is a single study and relies on imputed change SDs
Adverse events	Not pooled because adverse events were not systematically reported in most studies	Under-reported across trials	Very low	Harm, discomfort, and treatment burden remain insufficiently assessed
Pain, respiratory, postural, thoracic/rib-cage, pelvic, balance, and gait outcomes	Not pooled; absent, inconsistently reported, or not extractable in add-on-to-bracing comparisons	Sparse or no extractable add-on-to-bracing evidence	Very low/not estimable	Monitoring and research priorities; not established effects of Schroth plus bracing in this review

Schroth or Schroth-based exercises plus bracing compared with bracing alone for adolescents with idiopathic scoliosis.

## Discussion

### Principal findings

This review examined whether Schroth or explicitly Schroth-based exercises provide incremental benefits when added to bracing. The primary endpoint/final-value continuous synthesis favored combined treatment for Cobb angle, while ATR results were directionally favorable but uncertain because of substantial heterogeneity, and important small-sample uncertainty. Change-score and imputed-SD analyses were supportive of sensitivity evidence. However, the certainty was very low for all outcomes, and the SRS-22 evidence was sparse and sensitivity-based. The findings support Schroth as a possible adjunct to bracing but do not prove that it changes the long-term natural history of AIS, prevents surgery, or compensates for inadequate brace wear.

### Comparison with previous reviews

Unlike broader reviews that rank multiple conservative options (14), this review focused on a narrower rehabilitation question: whether Schroth adds benefit when brace treatment is already prescribed. The direction of effect is consistent with the wider scoliosis-specific exercise literature (38–41); however, the add-on evidence remains smaller and less stable because it depends on comparable brace dose, comparable follow-up, and complete reporting of endpoint/final-value and change-score variability. This distinction is important for rehabilitation medicine because a positive exercise signal in mixed conservative-care networks does not automatically indicate whether an already-braced adolescent should add a supervised Schroth program.

This review adds focus by separating Schroth as an add-on to bracing from exercise versus usual care, exercise alone, mixed exercise schools, reduced brace-dose designs, imputed SD analyses, and abstract-limited evidence. Treatment can be plausible and useful within multimodal conservative care while still lacking high-certainty evidence for preventing progression to surgical thresholds or improving patient-reported outcomes.

Therefore, the most defensible interpretation is cautiously supportive rather than confirmatory. The observed Cobb and ATR signals suggest that structured Schroth training may add measurable short-term benefits to brace-based care. However, the evidence remains too fragile to support claims that Schroth reliably changes skeletal-maturity outcomes, permits reduced brace wear in high-risk patients, or lowers operative treatment rates. This restrained interpretation is especially important in AIS, where baseline growth potential, curve magnitude, curve pattern, and brace adherence can dominate the prognosis.

### Clinical interpretation for rehabilitation practice

From a scoliosis-care perspective, Schroth and bracing should be understood as complementary rather than interchangeable. Bracing addresses the risk of growth-related mechanical progression, whereas Schroth trains active correction, trunk control, rotational breathing, body awareness, and daily postural strategies. This distinction is clinically relevant because Schroth may support brace-based rehabilitation without replacing the mechanical role of prescribed brace wear.

In a growing adolescent with meaningful progression risk, Schroth should not be considered a replacement for the prescribed brace treatment. A more defensible message is that Schroth may support brace-based care by improving active correction capacity, trunk symmetry, breathing, and rib-cage expansion strategies, comfort with corrected postures, and self-management during a demanding treatment course. For patients with larger curves, substantial growth remaining, poor in-brace correction, or documented progression, reducing brace wear on the assumption that exercise will compensate would require stronger evidence than that currently available.

This review also reinforces the need to distinguish a “pure add-on” strategy from the brace-dose reduction. A reduced-brace regimen tests a different question from Schroth, added to comparable bracing, even if both are relevant to shared decision-making. Families may reasonably ask whether exercise can make brace treatment easier, but a trial that lowers brace hours while adding exercise cannot isolate the incremental effect of Schroth over standard brace care. Future trials should state whether the goal is an additive benefit, brace-dose reduction, or improved tolerance of standard bracing.

### Beyond Cobb angle: bracing, active self-correction, and whole-chain rehabilitation

The clinical question is broader than whether an exercise program reduces the radiographic angle by a few degrees. Bracing can be beneficial by providing sustained external correction during growth, but it can also change how an adolescent moves, breathes, sleeps, and participates in daily activities. For some patients, brace treatment may affect chest-wall comfort, rib prominence, trunk mobility, balance, gait, skin tolerance, pain, body image, and willingness to remain physically active. These domains are not secondary decorations in conservative AIS care, and they influence whether the prescribed dose is actually wearable.

Schroth training is mechanistically attractive because it targets active three-dimensional self-correction rather than passive containment. Rotational angular breathing, axial elongation, derotation, stabilization, and daily-life transfer may plausibly influence thoracic expansion strategies, visible trunk asymmetry, rib-cage mechanics, postural control, and confidence during brace-based treatment (1, 12, 13, 15, 39, 52). Related exercise and biomechanical literature also support attention to surface topography, pelvic rotation, trunk-pelvis coordination, balance, gait, and lower-limb alignment when interpreting AIS rehabilitation (46–53).

However, the present review cannot claim that Schroth plus bracing improves broader outcomes. Within the included add-on-to-bracing studies, pain, respiratory function, thoracic or rib-cage mobility, detailed posture, pelvic alignment, balance, gait, and participation outcomes were too sparse or inconsistently reported for quantitative synthesis. These factors should, therefore, be used to deepen clinical reasoning and define future outcome sets, not to overstate the current evidence.

### Interpretation of radiographic and trunk-asymmetry outcomes

The pooled Cobb angle estimates suggested an additional improvement of approximately 3°–6°, depending on the analysis. This magnitude can be clinically meaningful near the common 5° progression thresholds or management boundaries. Nevertheless, Cobb measurement variability is not trivial, and small between-group differences should be interpreted in light of the known radiographic measurement error (42). Curve pattern, growth velocity, brace design, in-brace correction, radiograph timing, and adherence can all influence a difference of this magnitude. The mean Cobb change also does not necessarily indicate whether an individual avoided progression, crossed a surgical threshold, or maintained correction through brace weaning. Therefore, radiographic signals are promising but not definitive.

ATR is clinically relevant because it reflects visible trunk rotation, which adolescents and families often notice directly, and has long been used in scoliosis screening and surface assessment (43). Improvements in ATR may indicate better active postural control and surface symmetry. However, heterogeneity was substantial, and ATR can be influenced by the curve pattern, flexibility, examiner technique, out-of-brace timing, and the patient’s ability to perform active self-correction. ATR should be interpreted as an important complementary outcome rather than a surrogate for radiographic stabilization or surgical-risk reduction.

The substantial heterogeneity in Cobb angle and ATR syntheses probably reflects several clinical and methodological differences rather than a single inconsistent treatment effect. Baseline curve severity ranged from moderate to larger curves, skeletal maturity and growth potential differed, and curve pattern or flexibility was not consistently stratified. Brace approaches also varied, including nighttime Providence bracing, Cheneau-type or Cheneau-principle braces, thoracolumbosacral orthoses, three-dimensional printed braces, and studies with differing prescribed hours or unclear in-brace corrections. The exercise dose ranged from short supervised programs to daily home practice and 12-month supervised treatment, with variable therapist training, home-practice monitoring, and adherence support. Follow-up timing, endpoint versus change-score reporting, imputed variance data, and high or serious risk of bias further limited the exchangeability. Because each meta-analysis included a few studies, these factors could not be reliably tested using subgroup analysis or meta-regression and should be interpreted qualitatively.

The most promising future radiographic analyses will move beyond mean change alone. Clinically interpretable outcomes should include the proportion of patients with progression of at least 5°, the proportion crossing prespecified treatment thresholds, the proportion requiring surgery or reaching a surgical curve magnitude, and maintenance of correction after brace weaning. Such outcomes better match how spine specialists and families make decisions, particularly when a patient is near the management boundary.

### Quality of life, adherence, and treatment burden

The SRS-22 finding should be treated as hypothesis-generating because it came from sparse sensitivity evidence with imputed-variance data. Schroth could plausibly affect agency, body awareness, spinal appearance, satisfaction, function, or pain during bracing (44, 45, 52); however, the present review could not establish a reliable patient-reported outcome benefit. Adherence and burden remain central to interpretation: motivated families with better access to specialist care may complete both brace and exercise regimens more consistently, whereas pain, discomfort, fatigue, travel burden, cost, school schedules, heat, sleep disruption, and body-image concerns may reduce both brace wear and exercise completion.

Adherence requires particular attention. The adherence and burden table shows that several studies prescribed intensive exercise and long brace wear, but few used objective brace sensors, quantified exercise adherence, or systematically recorded discomfort, time burden, withdrawals, and missing data. This weakens external validity because real-world rehabilitation success depends on the dose actually received, not only the prescribed dose. It also limits clinical translation: an apparently effective protocol may be difficult to reproduce if it requires frequent supervised sessions, daily home exercise, long travel time, and high family monitoring without reporting the number of participants who could sustain that workload.

### Safety and adverse-event reporting

Safety should be interpreted cautiously because adverse events were reported inconsistently. The absence of reported events does not indicate the absence of harm. Future trials should actively capture skin irritation, brace pressure, pain, fatigue, exercise-related soreness or symptom flare, respiratory discomfort, psychosocial burden, sleep effects, activity restriction, and reasons for non-adherence.

### Methodological issues

A key methodological issue is the separation of the pure add-on question from broader pragmatic strategies. The registered question was whether Schroth adds benefit to bracing when brace treatment is otherwise comparable. A study that reduces brace wear in the Schroth group while maintaining full-time bracing in the comparator tests a different question: whether an exercise-supported lower brace-dose strategy can perform comparably to standard bracing. This question is patient-centered and important, but it is indirect for estimating the incremental contribution of Schroth over brace treatment alone.

The evidence base is statistically and clinically fragile. Adding endpoint final-value analyses improved information use for Cobb angle and ATR but did not remove the limits created by small *k*, heterogeneous follow-up, incomplete change SDs, and limited exchangeability. Change-score analyses remain useful sensitivity evidence, while missing SDs, event counts, withdrawals, and harm counts require author clarification before a more definitive synthesis.

### Implications for practice and research

Clinically, Schroth or Schroth-based exercises may be offered as an adjunct to bracing when trained therapists are available, and patients and families can sustain the additional burden. Treatment goals should be explicit: improving active self-correction, trunk control, surface asymmetry, breathing mechanics, tolerance of corrected posture, and self-management while preserving the prescribed brace regimen. Clinicians should monitor both brace wear and exercise adherence and avoid promising quality of life, pain, respiratory, or activity-participation benefits that remain uncertain.

A practical clinical assessment should, therefore, extend beyond the Cobb angle alone. In addition to radiographic progression and ATR, clinicians can monitor visible posture and rib prominence, shoulder and pelvic balance, chest expansion or pulmonary symptoms when relevant, pain, brace-related skin or pressure problems, SRS-22 or SRS-22r domains, physical activity participation, balance, gait, and patient-reported treatment burden. These measures help determine whether the combined regimen is feasible and whether the patient is gaining active control rather than merely receiving a larger treatment dose.

For clinical translation, an adjunctive program may need to preserve Schroth’s curve-specific aims while screening for modifiable whole-body factors, such as pelvic obliquity, apparent or structural leg-length inequality, hip and knee rotational asymmetry, foot loading, single-leg balance, trunk-pelvis dissociation, gait symmetry, respiratory strategy, and performance under vestibular or somatosensory challenge. Addressing these features may improve functional confidence, reduce perceived treatment burden, and support adherence; however, this remains a research hypothesis unless trials prospectively measure these outcomes together with the Cobb angle, ATR, brace wear, exercise dose, harm, and patient-reported function.

Next-generation randomized trials should be pragmatic and tightly monitored. Groups should receive the same prescribed brace dose and adjustment pathway, objective brace-wear sensors, transparent exercise logs, prespecified progression and surgery outcomes, adverse-event reporting, and complete SD/event-count reporting. Trials should also report why participants withdrew, missed visits, reduced brace wear, or stopped exercising. These feasibility data are not secondary decorations for rehabilitation interventions; they determine whether the protocol can be implemented outside specialist centers, whether benefits are attributable to exercise rather than cointervention intensity, and whether families experience the combined regimen as acceptable over months or years.

The core outcome set should be prespecified and include progression of at least 5°, crossing a surgical or treatment-escalation threshold, surgery recommendation or surgery, Cobb angle change, ATR, posture or surface-topography measures, chest expansion or pulmonary function where relevant, validated pain or function measures, SRS-22 or SRS-22r, brace adherence, exercise dose/adherence, treatment burden, adverse events, withdrawals, opportunity costs, and sex/gender-stratified reporting. Randomization should be stratified by progression risk, and analyses should report both intention-to-treat and adherence-informed estimates without allowing adherence-defined exclusions to drive the main results. Trials should also report on therapist credentialing, fidelity to Schroth principles, home-practice dose, cointerventions, and reasons for discontinuation. These details are essential because Schroth is not a single exercise but a skill-based therapist-mediated rehabilitation approach.

### Strengths and limitations

This review had several strengths: a focused PROSPERO-registered question, inclusion of Chinese and English evidence, duplicate full-text assessment, separate appraisal of randomized and non-randomized evidence, transparent sensitivity analyses, and explicit documentation of search custody. Limitations include a lack of Scopus and SinoMed/CBM access, partial CENTRAL capture because full export/authentication was unavailable, incomplete raw-export availability for some sources, very low certainty, sparse progression data, and weak reporting of adherence, harm, burden, patient-reported outcomes, pain, respiratory function, thoracic or rib-cage mobility, posture, pelvic alignment, gait, balance, and physical-activity participation. Access-attempt logs, rerun strategies, source-level custody manifests, available export/deduplication deposits, and PRESS-style self-audit materials are provided in Supplementary Data Sheet 1; however, these materials mitigate rather than eliminate search limitations.

A source-type limitation is that the comprehensive search identified publicly archived theses and abstract-limited reports, alongside peer-reviewed journal articles. These records improved search transparency but increased uncertainty regarding reporting completeness and source classification. Accordingly, the primary conclusions were anchored in the prespecified randomized endpoint/final-value analyses, while source-sensitive evidence was presented as sensitivity or exploratory material.

## Conclusion

Very low-certainty evidence suggests that Schroth or explicitly Schroth-based exercises, when added to bracing, may improve the short-term Cobb angle compared with bracing alone. For ATR, quality of life, pain, respiratory function, posture, chest-wall or rib-cage mobility, pelvic alignment, balance, gait, and other patient-prioritized outcomes, the available add-on-to-bracing evidence was too sparse, heterogeneous, or incompletely reported to determine whether additional benefits exist; this uncertainty should not be interpreted as evidence that combined bracing and exercise is ineffective for all AIS patients. Evidence is also insufficient for progression, surgery, durability after brace weaning, harm, adherence, and treatment burden. Schroth is best viewed as a monitored adjunct to prescribed bracing and not as a substitute for brace treatment. The most useful next evidence will combine radiographic endpoints with adherence, safety, treatment burden, postural and respiratory outcomes, whole-chain function, and patient-prioritized participation outcomes, so that clinicians can judge both effectiveness and feasibility in ordinary rehabilitation pathways. Until then, counseling should remain balanced, practical, individualized, and explicit, while being transparent about uncertainty, feasibility, and family preferences.

## Data Availability

The original contributions presented in this study are included in the article/Supplementary Material, further inquiries can be directed to the corresponding author.

## Supplementary material

SUPPLEMENTARY DATA SHEET 1 Supplementary Material includes PRISMA 2020 and PRISMA-S checklists, protocol-deviation summary, full search strategies, search archive and access-attempt logs, raw-export custody materials, PRESS-style search self-audit, full-text exclusion table, endpoint/final-value and sensitivity-analysis materials, statistical datasets and analysis scripts, adherence, exercise-dose, withdrawal, adverse-event, treatment-burden, missing-data, author-contact, reference-audit materials, and supplementary figures.
