# Supplementary material for: Schroth exercises added to bracing for adolescent idiopathic scoliosis: a systematic review and meta-analysis
Source: Front Sports Act Living. 2026 Sep 23;8:1919698. doi: 10.3389/fspor.2026.1919698 (PMC13642753; doi:10.3389/fspor.2026.1919698)
Supplement: Supplementary file 1 [file Datasheet1.zip › Frontiers_Sports_Active_Living_submission_ready_2026-06-25_Frontiers_guidelines_polished/supplementary_material/sup_fig5_cobb_sd_sensitivity.pdf]

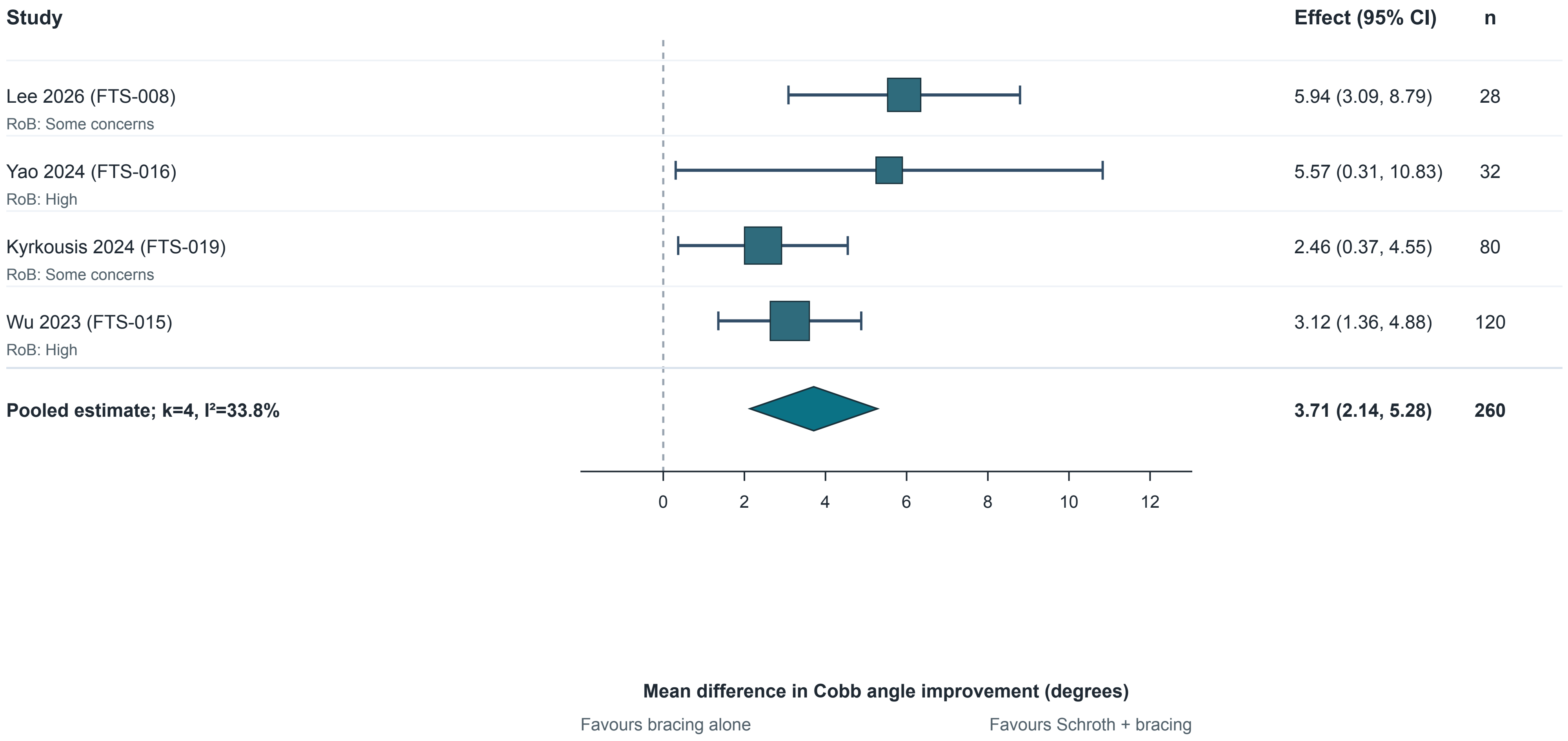
Study
Effect (95% CI)
n
Lee 2026 (FTS-008)
RoB: Some concerns
5.94 (3.09, 8.79)
28
Yao 2024 (FTS-016)
RoB: High
5.57 (0.31, 10.83)
32
Kyrkousis 2024 (FTS-019)
RoB: Some concerns
2.46 (0.37, 4.55)
80
Wu 2023 (FTS-015)
RoB: High
3.12 (1.36, 4.88)
120
Pooled estimate; k=4, I²=33.8%
3.71 (2.14, 5.28)
260
0
2
4
6
8
10
12
Mean difference in Cobb angle improvement (degrees)
Favours bracing alone
Favours Schroth + bracing
