# Supplementary material for: Schroth exercises added to bracing for adolescent idiopathic scoliosis: a systematic review and meta-analysis
Source: Front Sports Act Living. 2026 Sep 23;8:1919698. doi: 10.3389/fspor.2026.1919698 (PMC13642753; doi:10.3389/fspor.2026.1919698)
Supplement: Supplementary file 1 [file Datasheet1.zip › Frontiers_Sports_Active_Living_submission_ready_2026-06-25_Frontiers_guidelines_polished/supplementary_material/sup_fig1_rob2.pdf]

Supplementary Figure 1. RoB 2 traffic-light plot

Study	D1	D2	D3	D4	D5	Overall
Shi 2022 (FTS-001)	?	?	+	?	?	?
Liang 2023 (FTS-002)	?	?	?	?	?	?
Liu 2026 (FTS-003)	?	?	+	?	?	?
Lee 2026 (FTS-008)	?	?	+	+	?	?
Wu 2024 (FTS-014)	?	?	?	?	!	!
Wu 2023 (FTS-015)	?	?	?	?	!	!
Yao 2024 (FTS-016)	?	?	!	?	?	!
Kyrkousis 2024 (FTS-019)	?	+	+	+	?	?
Meng 2026 (FTS-021)	?	?	?	?	+	?

+ Low   ? Some concerns / Moderate   ! High / Serious

D1: d1 randomization; D2: d2 deviations; D3: d3 missing data; D4: d4 measurement; D5: d5 reported result
