# Supplementary material for: Schroth exercises added to bracing for adolescent idiopathic scoliosis: a systematic review and meta-analysis
Source: Front Sports Act Living. 2026 Sep 23;8:1919698. doi: 10.3389/fspor.2026.1919698 (PMC13642753; doi:10.3389/fspor.2026.1919698)
Supplement: Supplementary file 1 [file Datasheet1.zip › Frontiers_Sports_Active_Living_submission_ready_2026-06-25_Frontiers_guidelines_polished/supplementary_material/supp_endpoint_final_value_analysis.docx]

**Supplementary endpoint final-value analysis**

This file documents the primary continuous synthesis using raw post-intervention/final group means and SDs within prespecified clinically comparable follow-up and brace-dose contexts where available. Change-score analyses remain sensitivity evidence because many studies did not report SDs of change. Positive MDs favour Schroth or Schroth-based exercises plus bracing.

**Meta-analysis summary**

| **Outcome** | **Studies** | **Participants** | **Pooled MD** | **95% CI** | **HK CI** | **I2** |
| --- | --- | --- | --- | --- | --- | --- |
| Cobb angle | 4 | 330 | 3.78 | 2.27 to 5.29 | 1.84 to 5.71 | 69.9% |
| ATR | 3 | 220 | 1.82 | 0.16 to 3.48 | -1.18 to 4.83 | 92.4% |

Hartung-Knapp intervals are shown as small-sample sensitivity intervals and should be interpreted cautiously because k was 3-4.

**Endpoint rows**

| **Study** | **Outcome** | **Time** | **n I/C** | **Mean I/C** | **SD I/C** | **MD** | **Role** |
| --- | --- | --- | --- | --- | --- | --- | --- |
| Shi 2022 [10] | Cobb angle | 24 weeks | 51/50 | 24.89/27.42 | 2.07/2.39 | 2.53 | Primary endpoint |
| Liang 2023 [11] | Cobb angle | 6 months | 55/55 | 20.34/25.24 | 3.23/3.44 | 4.90 | Primary endpoint |
| Liu 2026 [12] | Cobb angle | 24 weeks | 19/20 | 26.03/31.07 | 5.95/8.17 | 5.04 | Primary endpoint |
| Kyrkousis 2024 [22] | Cobb angle | 12 months | 40/40 | 27.8/31.48 | 6.2/2.66 | 3.68 | Primary endpoint |
| Shi 2022 [10] | ATR | 24 weeks | 51/50 | 3.82/4.51 | 1.09/1.16 | 0.69 | Primary endpoint |
| Liu 2026 [12] | ATR | 24 weeks | 19/20 | 6.21/8.0 | 1.99/2.25 | 1.79 | Primary endpoint |
| Kyrkousis 2024 [22] | ATR | 12 months | 40/40 | 7.2/10.25 | 1.84/1.82 | 3.05 | Primary endpoint |
| Lee 2026 [14] | Cobb angle | 12 weeks | 21/21 | 27.79/28.24 | / |  | Supportive/sensitivity |
| Meng 2026 [23] | Cobb angle | 12 months | 25/22 | 24.8/26.31 | 10.66/8.02 | 1.51 | Supportive/sensitivity |
| Zapata 2023 [15] | Curve magnitude | Skeletal maturity/final follow-up | 37/37 | 25.0/32.0 | 11.0/14.0 | 7.00 | Supportive/sensitivity |
