# Supplementary material for: Schroth exercises added to bracing for adolescent idiopathic scoliosis: a systematic review and meta-analysis
Source: Front Sports Act Living. 2026 Sep 23;8:1919698. doi: 10.3389/fspor.2026.1919698 (PMC13642753; doi:10.3389/fspor.2026.1919698)
Supplement: Supplementary file 1 [file Datasheet1.zip › Frontiers_Sports_Active_Living_submission_ready_2026-06-25_Frontiers_guidelines_polished/supplementary_material/prisma_checklist.docx]

**PRISMA 2020 checklist - completed reporting map**

Manuscript: Schroth exercises added to bracing for adolescent idiopathic scoliosis: systematic review and meta-analysis. Locations are given by section because final page numbers may change after journal typesetting.

| **Item** | **Topic** | **Reported location** | **Comment** |
| --- | --- | --- | --- |
| 1 | Title | Title page; manuscript title | Identifies the report as a systematic review and meta-analysis. |
| 2 | Abstract | Abstract | Structured abstract includes background, objective, methods, results, conclusions, and registration. |
| 3 | Rationale | Introduction, paragraphs 1-4 | Explains AIS, bracing, Schroth mechanisms, and need for a focused add-on review. |
| 4 | Objectives | Abstract Objective; Introduction final paragraph | States the comparison of Schroth plus bracing versus bracing alone. |
| 5 | Eligibility criteria | Methods: Eligibility criteria | Population, intervention, comparator, study designs, and exclusions reported. |
| 6 | Information sources | Methods: Search and study selection; reporting_support/search_strategy_archive | Databases, trial registries, Chinese databases, and supplementary citation searching reported. |
| 7 | Search strategy | reporting_support/search_strategy_archive; Supplementary material plan | Search strings and search logs are included as support files. |
| 8 | Selection process | Methods: Search and study selection | Two-reviewer full-text assessment and consensus process reported. |
| 9 | Data collection process | Methods: Data extraction and risk of bias | Two-reviewer extraction of study, intervention, comparator, and outcome details reported. |
| 10a | Data items - outcomes | Methods: Data extraction; Outcomes and synthesis | Primary and secondary outcomes listed. |
| 10b | Data items - variables | Methods: Data extraction and risk of bias | Participant, brace, exercise-dose, follow-up, adherence, and funding/conflict variables reported. |
| 11 | Risk-of-bias assessment | Methods: Data extraction and risk of bias; Results: Risk of bias | RoB 2 for studies reported as randomized comparative trials and ROBINS-I for non-randomized studies. |
| 12 | Effect measures | Methods: Outcomes and synthesis | Mean differences for continuous outcomes and risk ratios for dichotomous outcomes. |
| 13a | Synthesis eligibility | Methods: Outcomes and synthesis | Randomized and non-randomized evidence synthesized separately; no-imputation primary dataset specified. |
| 13b | Data preparation | Methods: Outcomes and synthesis | Direction of continuous and dichotomous effects reported; SD-imputation formula reported. |
| 13c | Tabulation and visual display | Table 1; Table 2; Figures 1-6 | Study characteristics, SoF table, PRISMA flow, RoB, and forest plots provided. |
| 13d | Synthesis methods | Methods: Outcomes and synthesis | Random-effects inverse-variance DerSimonian-Laird approach and heterogeneity statistics reported. |
| 13e | Heterogeneity exploration | Results: Cobb angle; Angle of trunk rotation; Discussion | Heterogeneity and sensitivity analyses interpreted. |
| 13f | Sensitivity analyses | Methods: Outcomes and synthesis; Results: Cobb angle and ATR | Imputed-SD and unequal-brace-dose sensitivity analyses reported. |
| 14 | Reporting-bias assessment | Methods: Outcomes and synthesis; Results: Certainty of evidence | Funnel plot/Egger threshold stated; not performed because no synthesis had at least 10 studies. |
| 15 | Certainty assessment | Methods: Certainty of evidence; Results: Certainty of evidence; Table 2 | GRADE approach and judgements reported. |
| 16a | Study selection results | Results: Study selection; Figure 1 | Records, reports sought, retrieved reports, included studies, and exclusion summary reported. |
| 16b | Excluded studies | Results: Study selection; Figure 1 | Full-text exclusion count and most common reason reported. |
| 17 | Study characteristics | Results: Study characteristics; Table 1 | Design, participants, brace, exercise, comparator, follow-up, and outcomes summarized. |
| 18 | Risk of bias in studies | Results: Risk of bias; Figure 2; Supplementary Figures 1-2 | Overall and detailed RoB judgements provided. |
| 19 | Results of individual studies | Table 1; Figures 3-6; supplementary data files | Individual study contributions visible in forest plots and extraction files. |
| 20a | Results of syntheses | Results: Curve progression, Cobb angle, ATR, QoL/adverse events | Pooled estimates, confidence intervals, and participant counts reported. |
| 20b | Statistical heterogeneity | Results: Cobb angle; ATR | Heterogeneity interpreted where synthesis was possible. |
| 20c | Sensitivity analyses | Results: Cobb angle; ATR | Different imputed-SD correlations and pragmatic sensitivity analysis reported. |
| 20d | Reporting-bias results | Results: Certainty of evidence | Formal small-study-effect tests not performed due to too few studies. |
| 21 | Certainty of evidence | Results: Certainty of evidence; Table 2 | Very-low certainty for all critical and important outcomes explained. |
| 22 | Discussion - interpretation | Discussion: Principal findings; Clinical interpretation | Findings interpreted cautiously against clinical context and evidence limitations. |
| 23a | Discussion - limitations of evidence | Discussion: Methodological issues; Strengths and limitations | Risk of bias, SD imputation, adherence, heterogeneity, follow-up, and harms reporting discussed. |
| 23b | Discussion - limitations of review process | Discussion: Strengths and limitations | Evidence separation and transparent SD-imputation approach discussed. |
| 23c | Discussion - implications | Discussion: Implications for practice and research; Conclusion | Clinical and trial-design implications reported. |
| 24a | Registration | Abstract; title page; Methods: Registration and reporting | PROSPERO CRD420261397961 reported, including at the end of the abstract. |
| 24b | Protocol access | Methods: Registration and reporting; PROSPERO record | Protocol registration reported; full protocol not separately written. |
| 24c | Protocol amendments | Methods; Supplementary material plan | Protocol-related operational deviations are explicitly reported: Scopus and SinoMed/CBM were not searched because access was unavailable; CENTRAL could only be partially captured/exported; Google Scholar was used only for citation checking. A final analytic clarification/deviation stratified continuous outcomes by clinical directness, follow-up compatibility, data completeness, and brace-dose comparability, demoting cross-stratum pooling to sensitivity or exploratory evidence. See Methods, Supplementary Search Strategy, PRISMA-S checklist, and protocol-deviation summary. |
| 25 | Support | Title page; Declarations | Funding sources and funder role reported. |
| 26 | Competing interests | Title page; Declarations | No competing interests declared. |
| 27 | Availability of data/code/materials | Declarations; supplementary_files/ | Extracted data and analysis files included or available from the project archive. |
