# Supplementary material for: Schroth exercises added to bracing for adolescent idiopathic scoliosis: a systematic review and meta-analysis
Source: Front Sports Act Living. 2026 Sep 23;8:1919698. doi: 10.3389/fspor.2026.1919698 (PMC13642753; doi:10.3389/fspor.2026.1919698)
Supplement: Supplementary file 1 [file Datasheet1.zip › Frontiers_Sports_Active_Living_submission_ready_2026-06-25_Frontiers_guidelines_polished/supplementary_material/sup_fig6_atr_sd_sensitivity.pdf]

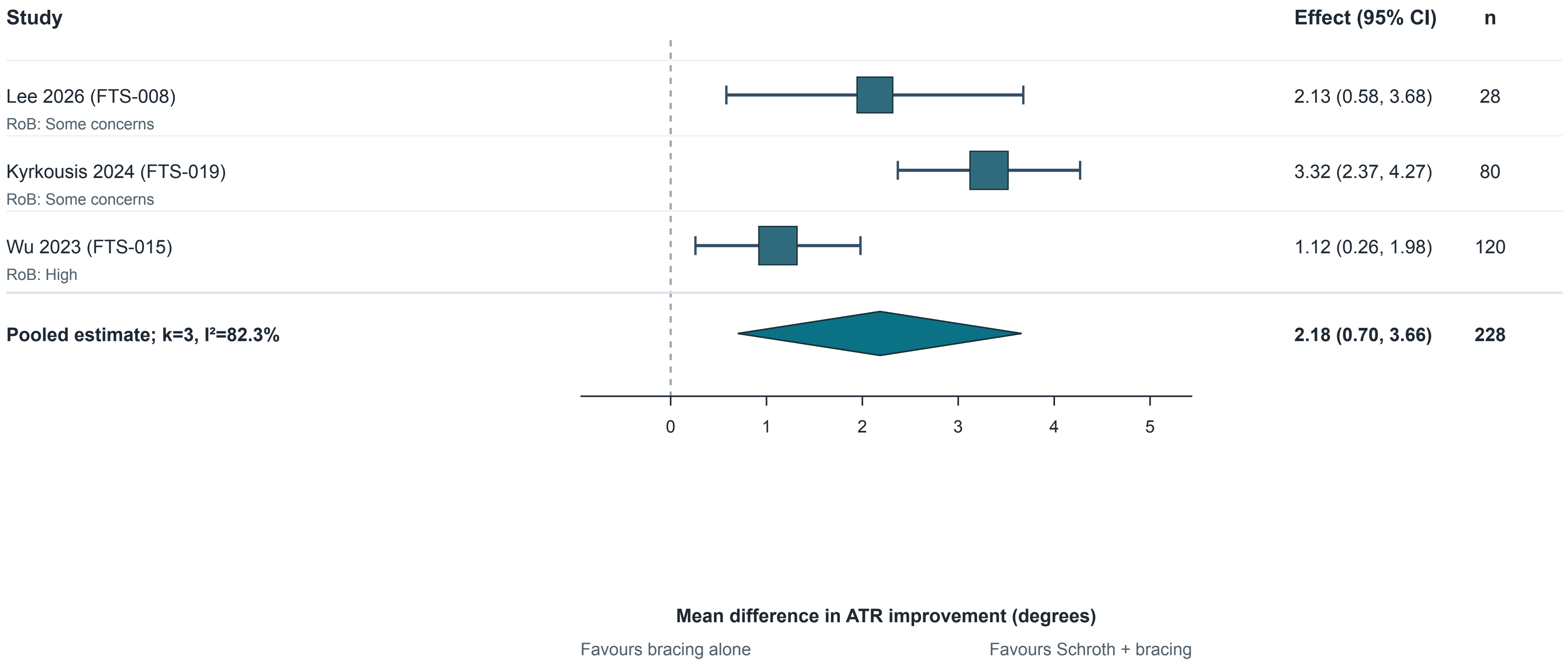

Study
Effect (95% CI)
n
Lee 2026 (FTS-008)
RoB: Some concerns
2.13 (0.58, 3.68)
28
Kyrkousis 2024 (FTS-019)
RoB: Some concerns
3.32 (2.37, 4.27)
80
Wu 2023 (FTS-015)
RoB: High
1.12 (0.26, 1.98)
120
Pooled estimate; k=3, I²=82.3%
2.18 (0.70, 3.66)
228
0
1
2
3
4
5
Mean difference in ATR improvement (degrees)
Favours bracing alone
Favours Schroth + bracing
