# Supplementary material for: Schroth exercises added to bracing for adolescent idiopathic scoliosis: a systematic review and meta-analysis
Source: Front Sports Act Living. 2026 Sep 23;8:1919698. doi: 10.3389/fspor.2026.1919698 (PMC13642753; doi:10.3389/fspor.2026.1919698)
Supplement: Supplementary file 1 [file Datasheet1.zip › Frontiers_Sports_Active_Living_submission_ready_2026-06-25_Frontiers_guidelines_polished/supplementary_material/sup_fig4_cobb_change_noimp_sensitivity.pdf]

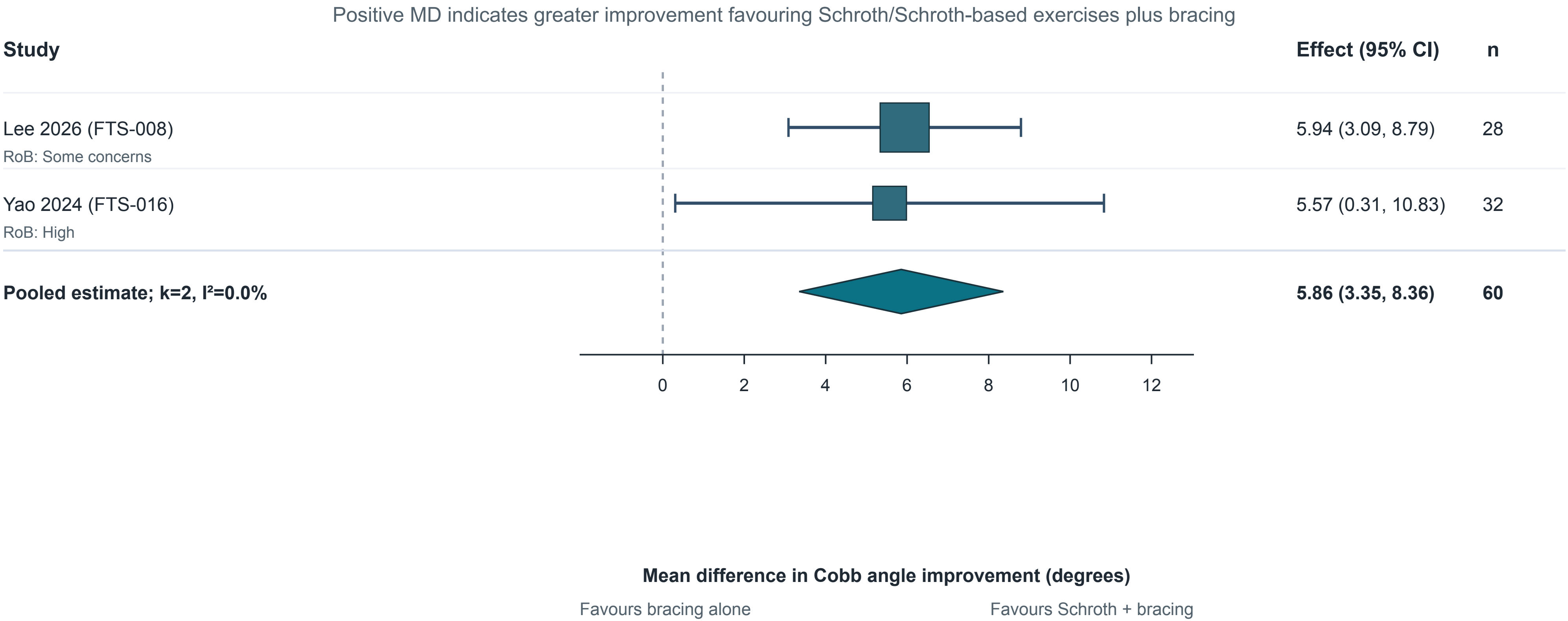
Positive MD indicates greater improvement favouring Schroth/Schroth-based exercises plus bracing
Study
Effect (95% CI)
n
Lee 2026 (FTS-008)
RoB: Some concerns
5.94 (3.09, 8.79)
28
Yao 2024 (FTS-016)
RoB: High
5.57 (0.31, 10.83)
32
Pooled estimate; k=2, I²=0.0%
5.86 (3.35, 8.36)
60
0
2
4
6
8
10
12
Mean difference in Cobb angle improvement (degrees)
Favours bracing alone
Favours Schroth + bracing
