# Supplementary material for: Schroth exercises added to bracing for adolescent idiopathic scoliosis: a systematic review and meta-analysis
Source: Front Sports Act Living. 2026 Sep 23;8:1919698. doi: 10.3389/fspor.2026.1919698 (PMC13642753; doi:10.3389/fspor.2026.1919698)
Supplement: Supplementary file 1 [file Datasheet1.zip › Frontiers_Sports_Active_Living_submission_ready_2026-06-25_Frontiers_guidelines_polished/supplementary_material/protocol_deviations.docx]

# Protocol deviations and operational limitations

This summary distinguishes operational search limitations and final analytic hierarchy clarifications from the registered PICO.

| **Deviation/clarification** | **Type** | **Reason** | **How handled** |
| --- | --- | --- | --- |
| Scopus not searched | Information source/access | Scopus was prespecified but institutional access was unavailable during the final search window. | Listed as not searched; considered in limitations as possible missed records. |
| SinoMed/CBM not searched | Information source/access | SinoMed/CBM was prespecified but institutional access was unavailable during the final search window. | Listed as not searched; considered in limitations as possible missed Chinese biomedical records. |
| CENTRAL partial capture/export | Information source/export | CENTRAL was searched, but full export/authentication to complete CENTRAL content was unavailable. | Visible metadata/counts archived; described as partial capture and operational limitation. |
| Google Scholar status | Supplementary source reproducibility | Google Scholar was not treated as a completed database because stable reproducible bulk export was unavailable. | Used only for citation checking; not counted as a completed database. |
| Raw exports not all packaged | Search archive auditability | Not all raw database exports and deduplication files are included in the public submission archive. | Manifest lists source status, retained/export status, substitute evidence, and availability on reasonable request. |
| Reports not retrieved | Study retrieval | Seven sought reports/records were not retrieved. | Reported in PRISMA flow, study selection, limitations, and full-text/retrieval documentation. |
| Primary continuous synthesis hierarchy | Analysis hierarchy | Continuous outcomes differed in follow-up window, brace context, and data completeness. | Primary continuous synthesis uses endpoint/final values within prespecified comparable follow-up and brace-dose contexts; change-score, imputed-SD, mixed-follow-up, abstract-limited, unequal-brace-dose, and non-randomized analyses are sensitivity or exploratory evidence. |
| Missing change SDs | Analysis hierarchy | Several studies lacked directly reported change SDs. | Change SDs were imputed only in exploratory sensitivity analyses at r=0.25, 0.50, and 0.75. |
| Abstract-limited Wu 2023/FTS-015 | Evidence certainty | Wu 2023/FTS-015 was available only as extractable abstract-level evidence with incomplete intervention/timing reporting. | Included only as exploratory high-uncertainty imputed-SD sensitivity evidence. |
| Unequal brace-dose Meng 2026/FTS-021 | Clinical directness | Meng 2026 compared Schroth plus lower prescribed brace dose with fuller brace dose in controls. | Kept separate as pragmatic indirect sensitivity evidence and not pooled with the primary endpoint/final-value synthesis. |
