# Supplementary material for: Schroth exercises added to bracing for adolescent idiopathic scoliosis: a systematic review and meta-analysis
Source: Front Sports Act Living. 2026 Sep 23;8:1919698. doi: 10.3389/fspor.2026.1919698 (PMC13642753; doi:10.3389/fspor.2026.1919698)
Supplement: Supplementary file 1 [file Datasheet1.zip › Frontiers_Sports_Active_Living_submission_ready_2026-06-25_Frontiers_guidelines_polished/supplementary_material/author_contact_missing_data_log_2026-06-06.docx]

**Author contact log for missing SDs, event counts, and harms/adherence data**

This file identifies missing information that should be requested from study authors. It is a prepared contact log/template and does not claim that emails were sent unless the authors later update the status column.

| **Study** | **Missing or unclear data requested** | **Purpose** | **Status in this revision** |
| --- | --- | --- | --- |
| Shi 2022 [10] | Change SDs for Cobb/ATR; adverse-event counts if collected | Allow change-score pooling and harm synthesis | Contact request template prepared; not marked sent |
| Liang 2023 [11] | Change SDs, ATR endpoint/change details, withdrawals, adverse events | Improve continuous and harm analyses | Contact request template prepared; not marked sent |
| Liu 2026 [12] | Change SDs for Cobb/ATR; adverse-event counts if collected | Allow change-score pooling and harm synthesis | Contact request template prepared; not marked sent |
| Kwan 2017 [13] | Withdrawals by group, raw endpoint/change SDs not in extractable tables, adherence details if available | Improve endpoint/change and attrition interpretation | Contact request template prepared; not marked sent |
| Lee 2026 [14] | Raw post-intervention means/SDs by group; event/adverse-event counts | Permit raw endpoint pooling and harm synthesis | Contact request template prepared; not marked sent |
| Zapata 2023 [15] | Adverse events and reasons for withdrawal/loss by group | Improve harms and missingness assessment | Contact request template prepared; not marked sent |
| Kastrinis 2026 [16] | Withdrawals, adverse events, and exact endpoint/change SDs if available | Improve harm and continuous evidence | Contact request template prepared; not marked sent |
| Wu 2024 [18] | Full report details, group sizes, endpoint/change means/SDs, withdrawals, adverse events | Resolve abstract-limited evidence | Contact request template prepared; not marked sent |
| Wu 2023 [19] | Full report details, group sizes, endpoint/change means/SDs, withdrawals, adverse events | Resolve abstract-limited evidence | Contact request template prepared; not marked sent |
| Yao 2024 [20] | Clarify final means/SDs vs change values, excluded participants, adverse events | Improve endpoint/change and missingness interpretation | Contact request template prepared; not marked sent |
| Kong 2023 [21] | Withdrawals, adverse events, adherence denominator, raw endpoint/change SDs | Improve feasibility and harm interpretation | Contact request template prepared; not marked sent |
| Meng 2026 [23] | Adverse-event counts and additional event counts if collected | Improve harm synthesis | Contact request template prepared; not marked sent |
