# Supplementary material for: Schroth exercises added to bracing for adolescent idiopathic scoliosis: a systematic review and meta-analysis
Source: Front Sports Act Living. 2026 Sep 23;8:1919698. doi: 10.3389/fspor.2026.1919698 (PMC13642753; doi:10.3389/fspor.2026.1919698)
Supplement: Supplementary file 1 [file Datasheet1.zip › Frontiers_Sports_Active_Living_submission_ready_2026-06-25_Frontiers_guidelines_polished/supplementary_material/sup_fig2_robinsi.pdf]

Supplementary Figure 2. ROBINS-I traffic-light plot

Study	D1	D2	D3	D4	D5	D6	D7	Overall
Kwan 2017 (FTS-007)	!	!	+	?	?	+	?	!
Zapata 2023 (FTS-009)	!	?	+	?	!	?	?	!
Kastrinis 2026 (FTS-010)	!	!	?	?	?	?	?	!
Fang 2022 (FTS-012)	!	?	+	?	?	?	?	!
Kong 2023 (FTS-017)	!	!	?	?	!	?	?	!

+ Low ? Some concerns / Moderate ! High / Serious

D1: d1 confounding; D2: d2 selection; D3: d3 classification; D4: d4 deviations; D5: d5 missing data; D6: d6 measurement; D7: d7 reported result
