# Supplementary material for: Schroth exercises added to bracing for adolescent idiopathic scoliosis: a systematic review and meta-analysis
Source: Front Sports Act Living. 2026 Sep 23;8:1919698. doi: 10.3389/fspor.2026.1919698 (PMC13642753; doi:10.3389/fspor.2026.1919698)
Supplement: Supplementary file 1 [file Datasheet1.zip › Frontiers_Sports_Active_Living_submission_ready_2026-06-25_Frontiers_guidelines_polished/supplementary_material/prisma_s_checklist.docx]

# PRISMA-S search reporting checklist and audit map

This checklist maps the submitted search documentation to PRISMA-S reporting domains relevant to this review.

| **PRISMA-S domain** | **Reported location** | **Current package evidence** |
| --- | --- | --- |
| Database names and platforms | Supplementary Search Strategy; PRISMA-S checklist | Completed sources listed with platform/interface: PubMed/MEDLINE, Embase, CENTRAL, CINAHL Complete, Web of Science Core Collection, CNKI, Wanfang, VIP, PEDro, ClinicalTrials.gov, WHO ICTRP, and citation checking. |
| Search dates | Supplementary Search Strategy; search log | Final search window 2026-05-18 to 2026-05-23; each source has a source-level search date in the log/manifest. |
| Full strategies | Supplementary Search Strategy; search archive | Core English strategy, database-specific strategy notes, PEDro short searches, Chinese database terms, and registry strategy are provided. |
| Limits and restrictions | Supplementary Search Strategy | No language or date restrictions at search stage; age/PICO restrictions applied during screening. |
| Non-database sources | Supplementary Search Strategy; manuscript Methods | ClinicalTrials.gov, WHO ICTRP, reference-list/forward-citation checking, and Google Scholar citation checking status are documented. |
| Unsearched prespecified sources | Manuscript Registration and reporting; Supplementary Search Strategy; protocol-deviation summary | Scopus and SinoMed/CBM were prespecified but not searched because institutional access was unavailable during the final search window. |
| Partial source capture | Manuscript Registration and reporting; Supplementary Search Strategy; protocol-deviation summary | CENTRAL was searched, but full export/authentication was unavailable; visible metadata and counts were archived. |
| Record management and deduplication | Search archive manifest; PRISMA identification counts | Record import, deduplication counts, and PRISMA count derivation are documented in the manifest and count file. |
| Raw exports and custody | Search archive manifest; README reproducibility | Raw exports are not all packaged; where export was available, files are retained by authors and available on reasonable request. Substitute evidence is listed for sources with limited export. |
| Protocol deviations | Protocol-deviation summary; manuscript Methods/Limitations | Unavailable database access, CENTRAL partial export, and final analytic stratification/demotion of clinically mixed pooling are explicitly reported. |
