# Supplementary material for: Schroth exercises added to bracing for adolescent idiopathic scoliosis: a systematic review and meta-analysis
Source: Front Sports Act Living. 2026 Sep 23;8:1919698. doi: 10.3389/fspor.2026.1919698 (PMC13642753; doi:10.3389/fspor.2026.1919698)
Supplement: Supplementary file 1 [file Datasheet1.zip › Frontiers_Sports_Active_Living_submission_ready_2026-06-25_Frontiers_guidelines_polished/supplementary_material/search_methods_mitigation_PRESS_repository_2026-06-06.docx]

**Search-methods mitigation, PRESS-style self-audit, and repository custody note**

This note documents mitigation for unavailable Scopus and SinoMed/CBM access, partial CENTRAL capture, raw-export/deduplication custody, and search peer-review readiness. It does not claim that Scopus or SinoMed/CBM were searched, nor that an external PRESS reviewer has endorsed the strategy.

**Scopus and SinoMed/CBM**

Access was unavailable during the final search window and the 2026-06-03 recheck. Access attempts, exact rerun strategies, and export templates are archived so that the searches can be rerun by a librarian or institutional account if access becomes available.

**CENTRAL**

CENTRAL was searched in the Trials tab. Counts were recorded, but full bulk export/authentication failed; visible metadata and search logs were archived. The review treats this as partial capture.

**Raw exports and deduplication**

Available raw exports, database exports, search logs, merged records, duplicate-removal logs, deduplicated records, and final screening/data-extraction workbooks were placed in the editor-facing repository deposit folder. A custody manifest identifies unavailable or partially captured sources.

**PRESS-style review**

A PRESS-style self-audit checklist was prepared to flag syntax, translation of the research question, synonyms, limits, deduplication, and unavailable-source issues. No external PRESS endorsement is claimed.
