# Supplementary material for: Schroth exercises added to bracing for adolescent idiopathic scoliosis: a systematic review and meta-analysis
Source: Front Sports Act Living. 2026 Sep 23;8:1919698. doi: 10.3389/fspor.2026.1919698 (PMC13642753; doi:10.3389/fspor.2026.1919698)
Supplement: Supplementary file 1 [file Datasheet1.zip › Frontiers_Sports_Active_Living_submission_ready_2026-06-25_Frontiers_guidelines_polished/supplementary_material/excluded_full_texts.docx]

# Supplementary Table. Full-text reports excluded or not counted separately

Reports were assessed independently by two reviewers. The table lists reports excluded after full-text assessment and one companion duplicate report retained for mapping but not counted as a separate included study.

| ID | Authors/year | Title | Decision/reason | Details |
| --- | --- | --- | --- | --- |
| FTS-004 | 姚鸣宇; 朱赫; 董亦直; 宋鑫越; 都亚新; 吴瑞霞; 祝勇 (2024) | 施罗斯PSSE联合3D打印支具治疗青少年特发性脊柱侧弯的疗效 | Companion/duplicate - do not count separately: Duplicate publication | PICO eligible, but highly likely same Yao 2024 cohort as FTS-016; use the fuller thesis report for extraction and keep this as companion evidence. |
| FTS-005 | 杨闪闪; 武改; 李丽; 王妍 (2025) | 脊柱侧弯矫形器联合施罗特疗法治疗小儿特发性脊柱侧凸的疗效观察 | Exclude: Wrong population | Pediatric/early-onset population; age range 7-12 and AIS adolescent subgroup is not extractable. |
| FTS-006 | 沈玄霖; 关月红; 范成功 (2018) | 色努矫形器结合施罗斯体操治疗青少年特发性脊柱侧弯的疗效 | Exclude: Wrong comparator | Comparator is Schroth exercise alone, not brace alone. |
| FTS-011 | Kuru Çolak T; Akçay B; Apti A; Çolak İ (2023) | The Effectiveness of the Schroth Best Practice Program and Chêneau-Type Brace Treatment in Adolescent Idiopathic Scoliosis: Long-Term Follow-Up Evaluation Results. | Exclude: Wrong comparator | Single-arm long-term follow-up of Schroth Best Practice plus Cheneau-type brace; no brace-alone comparator. |
| FTS-013 | 段隽 (2026) | 脊柱矫形器联合施罗特疗法对小儿特发性脊柱侧凸患者Cobb角、疼痛及生活质量的改善作用 | Exclude: Wrong population | Pediatric idiopathic scoliosis population; adolescent AIS subgroup is not clearly defined or extractable. |
| FTS-018 | Brauner, B.; Braunerné Árkai, J.; Boncz, I.; Molics, B.; Ács, P.; Tardi, P. (2022) | POSA340 Investigation of the Effect of Schroth Therapy and Brace Therapy on Quality of Life in Children with Idiopathic Scoliosis | Exclude: Wrong comparator | Conference abstract with n=8 single-group Schroth-based exercises plus Cheneau corset; no brace-alone comparator. |
| FTS-020 | (2023) | The Effect of Schroth Best Practice Exercises and Cheneau Brace Treatment on Perceptual and Cognitive Asymmetry in Adolescent Idiopathic Scoliosis With Thoracic Major Curve | Exclude: Wrong comparator | Pre-experimental AIS group receiving SBP plus Cheneau brace compared with healthy controls; no brace-alone comparator and primary outcome is auditory perceptual/cognitive asymmetry. |
| FTS-022 | 王民政; 吴凡; 许权; 曹端广; 张恒青 (2025) | 中医综合疗法结合施罗斯疗法加支具矫正治疗青少年特发性脊柱侧弯的临床研究 | Exclude: Wrong comparator | Three-arm trial compares Schroth plus brace, traditional Chinese medicine plus brace, and combined TCM plus Schroth plus brace; no brace-alone comparator and target effect is confounded by TCM co-intervention. |
| FTS-023 | Ohlendorf D; Schlegel C; Oremek GM; Holzgreve F; Wanke EM; Mauer-Grubinger C; Diaremes P; Hülstrunk C; Zabar O; Groneberg DA (2024) | No effects of four weeks of combined brace and Schroth therapy in moderate adolescent idiopathic scoliosis on plantar pressure distribution. | Exclude: Wrong comparator | Single-cohort combined brace plus Schroth inpatient study; no brace-alone comparator, and measured plantar pressure rather than prespecified clinical/radiographic outcomes. |
| FTS-024 | Schreiber S; Parent EC; Hill DL; Hedden DM; Moreau MJ; Southon SC (2019) | Patients with adolescent idiopathic scoliosis perceive positive improvements regardless of change in the Cobb angle - Results from a randomized controlled trial comparing a 6-month Schroth intervention added to standard care and standard care alone. SOSORT 2018 Award winner. | Exclude: Wrong comparator | RCT secondary analysis of Schroth plus standard care versus standard care alone; standard care is observation or bracing, and brace-treated subgroup is not separately extractable. |
