# Supplementary material for: Schroth exercises added to bracing for adolescent idiopathic scoliosis: a systematic review and meta-analysis
Source: Front Sports Act Living. 2026 Sep 23;8:1919698. doi: 10.3389/fspor.2026.1919698 (PMC13642753; doi:10.3389/fspor.2026.1919698)
Supplement: Supplementary file 1 [file Datasheet1.zip › Frontiers_Sports_Active_Living_submission_ready_2026-06-25_Frontiers_guidelines_polished/supplementary_material/supp_adherence_harms_burden.docx]

**Supplementary table. Brace adherence, exercise dose, withdrawals, adverse events, and treatment burden/source of missingness.**

NR, not reported. Values are extracted as reported; absence of adverse-event reporting should not be interpreted as absence of harm.

| **Study** | **Brace adherence** | **Exercise dose/adherence** | **Withdrawals/missingness** | **Adverse events** | **Burden/source of missingness** |
| --- | --- | --- | --- | --- | --- |
| Shi 2022 [10] | Prescribed >=22 h/day after 2-week adaptation; clinical follow-up; no objective sensor; actual hours NR. | Schroth 3 sessions/week, 1.5 h/session, 24 weeks; home exercise unclear; exercise adherence NR. | 0/51 vs 0/50 withdrawals reported. | NR. | High prescribed brace time plus supervised exercise; missing adherence and harm data from non-reporting. |
| Liang 2023 [11] | Prescribed >18 h/day from week 3 for 6 months; no objective monitoring; actual hours NR. | Schroth 3 sessions/week, 90 min/session, 6 months; adherence NR. | Withdrawals NR. | NR. | Substantial time commitment; missingness mainly from incomplete reporting. |
| Liu 2026 [12] | Prescribed >=20 h/day for 24 weeks; weekly phone/parent/therapist follow-up; no sensor; actual hours NR. | Schroth 5 sessions/week, 60 min/session, 24 weeks; home training on non-hospital days; quantitative adherence NR. | 0/19 vs 0/20 withdrawals reported. | NR. | Frequent supervised/home exercise; harm and quantitative adherence missing from non-reporting. |
| Kwan 2017 [13] | Minimum 18 h/day; clinical compliance rating; good compliance 70.8% vs 79.2%; no objective sensor. | Private sessions every 2 weeks for 8 weeks plus home exercise >=5 days/week; caregiver verification and therapist checklist. | Withdrawals NR. | No adverse effects noted. | Home program burden documented indirectly; withdrawal data missing. |
| Lee 2026 [14] | At least 18 h/day for 12 weeks; adherence not objectively monitored; actual hours NR. | Schroth 3 sessions/week, 50 min/session, 12 weeks; adherence not objectively monitored. | 0/21 vs 0/21; all participants analysed. | Potential risks discussed, but separate adverse-event counts NR. | Short but intensive program; harm data limited by non-reporting of counts. |
| Zapata 2023 [15] | Nighttime Providence brace; temperature sensor every 15 min; course wear 8.0 +/- 2.9 vs 7.3 +/- 2.7 h/night. | Home PSSE >=15 min/day, 5 days/week for >=1 year plus >=8 h outpatient PT; adherence 64% at 6 months and 51% at 1 year. | 21/58 vs 20/57 withdrew or were lost to follow-up. | NR. | Long home program; attrition and declining adherence are major external-validity concerns. |
| Kastrinis 2026 [16] | Full-time 18-22 h/day; self-reported compliance 86.3% vs 81.3% at 6 months. | Supervised twice/week, 1 h/session plus home 4 times/week, 30 min/session for 6 months; exercise adherence not separately quantified. | Withdrawals NR. | NR. | Combined supervised and home schedule; self-report adherence and missing withdrawals/harm counts. |
| Fang 2022 [17] | Prescribed >=20 h/day; logbooks with parent/therapist verification; actual 21.58 +/- 0.66 vs 20.92 +/- 0.79 h/day. | Supervised 3 days/week for 6 weeks plus daily 30-45 min home exercise; logbook adherence. | Lost-to-follow-up participants excluded; analysed cohort only. | Formal adverse-event counts NR; brace-related pain/HRQoL concerns discussed. | High brace dose and daily exercise; selection/missingness from excluding incomplete follow-up. |
| Wu 2024 [18] | NR in extractable abstract. | NR in extractable abstract. | NR. | NR. | Abstract-limited reporting. |
| Wu 2023 [19] | NR in extractable abstract. | NR in extractable abstract. | NR. | NR. | Abstract-limited reporting. |
| Yao 2024 [20] | After 2-week adaptation, >=18 h/day; eligibility/exclusion based on wear >=18 h/day; no objective sensor; actual hours NR. | Weeks 1-4: 2-3 sessions/week, 45-60 min; thereafter 2-3/week, 30-45 min with WeChat/therapist follow-up. | 4/20 vs 4/20 excluded as outlier/lost/ineligible for final analysis. | No formal adverse-event table; soreness after Schroth training mentioned. | Exclusions after allocation and non-objective adherence monitoring limit interpretability. |
| Kong 2023 [21] | Brace worn except sleeping/bathing; brace maker supervised daily wearing, weekly feedback; actual hours NR. | Intensive 5-7 sessions/week, 4-6 h/day for 2-7 weeks, then >=1 h/day daily-life training; quantitative adherence NR. | Withdrawals NR; inclusion required >=1 year treatment/training completion. | Treatment risk described as low; no adverse-event counts. | Very high exercise burden; completion-based inclusion may select adherent participants. |
| Kyrkousis 2024 [22] | Mean daily brace wear 18.6 +/- 1.97 vs 17.95 +/- 1.88 h/day; measure not clearly reported. | Schroth 3 sessions/week, 60 min/session for 12 months; missed sessions rescheduled; no participant missed >7 days. | ITT retained 40/group; 2 vs 1 missed 12-month measurement, plus 2 vs 2 stopped communication by 18 months. | No complications or adverse symptoms reported. | Long supervised program; follow-up communication loss after intervention. |
| Meng 2026 [23] | Unequal brace dose: 14-18 h/day plus Schroth vs 20-24 h/day control; actual 14.72 +/- 0.85 vs 21.22 +/- 1.15 h/day. | 4-5 weekly sessions initially; mean exercise 4.9 h/week; logs, checklists, videos, weekly outpatient review. | 1/26 vs 4/26 withdrew after randomization; final analysis 25 vs 22. | Not reported as adverse-event counts. | Pragmatic brace-dose reduction context; not pure add-on evidence; incomplete harm counts. |
