# Supplementary material for: Schroth exercises added to bracing for adolescent idiopathic scoliosis: a systematic review and meta-analysis
Source: Front Sports Act Living. 2026 Sep 23;8:1919698. doi: 10.3389/fspor.2026.1919698 (PMC13642753; doi:10.3389/fspor.2026.1919698)
Supplement: Supplementary file 1 [file Datasheet1.zip › Frontiers_Sports_Active_Living_submission_ready_2026-06-25_Frontiers_guidelines_polished/supplementary_material/supp_stat_sensitivity.docx]

# Supplementary statistical robustness table

Primary continuous synthesis used endpoint/final values within prespecified clinically comparable follow-up and brace-dose contexts where available. The analyses in this file are change-score, no-imputation, imputed-SD, mixed-follow-up, abstract-limited, or unequal-brace-dose sensitivity/exploratory evidence. Full analytic notes are provided in the accompanying CSV.

| **Analysis** | **Model** | **k** | **Studies** | **Effect (95% CI)** | **95% prediction interval** | **Heterogeneity** |
| --- | --- | --- | --- | --- | --- | --- |
| Cobb change-score/no-imputation sensitivity stratum: Lee 2026 | Single-study estimate (no pooling) | 1 | Lee 2026 (FTS-008) | 5.940 (3.087 to 8.793) | NA | tau2=0.0000; I2=0.0% |
| Cobb change-score/no-imputation sensitivity stratum: Yao 2024 | Single-study estimate (no pooling) | 1 | Yao 2024 (FTS-016) | 5.570 (0.306 to 10.834) | NA | tau2=0.0000; I2=0.0% |
| Cobb limited no-imputation sensitivity | Fixed-effect inverse-variance | 2 | Lee 2026 (FTS-008); Yao 2024 (FTS-016) | 5.856 (3.348 to 8.364) | NA | tau2=0.0000; I2=0.0% |
| Cobb limited no-imputation sensitivity | DerSimonian-Laird random-effects | 2 | Lee 2026 (FTS-008); Yao 2024 (FTS-016) | 5.856 (3.348 to 8.364) | -10.405 to 22.117 | tau2=0.0000; I2=0.0% |
| Cobb limited no-imputation sensitivity | REML random-effects | 2 | Lee 2026 (FTS-008); Yao 2024 (FTS-016) | 5.856 (3.348 to 8.364) | -10.405 to 22.117 | tau2=0.0000; I2=0.0% |
| Cobb limited no-imputation sensitivity | DerSimonian-Laird Hartung-Knapp | 2 | Lee 2026 (FTS-008); Yao 2024 (FTS-016) | 5.856 (-10.405 to 22.117) | -10.405 to 22.117 | tau2=0.0000; I2=0.0% |
| Cobb limited no-imputation sensitivity | REML Hartung-Knapp | 2 | Lee 2026 (FTS-008); Yao 2024 (FTS-016) | 5.856 (-10.405 to 22.117) | -10.405 to 22.117 | tau2=0.0000; I2=0.0% |
| Cobb pragmatic indirect: unequal brace dose | Single-study estimate (no pooling) | 1 | Meng 2026 (FTS-021) | 4.050 (1.880 to 6.220) | NA | tau2=0.0000; I2=0.0% |
| ATR change-score/no-imputation sensitivity stratum: Lee 2026 | Single-study estimate (no pooling) | 1 | Lee 2026 (FTS-008) | 2.130 (0.581 to 3.679) | NA | tau2=0.0000; I2=0.0% |
| ATR pragmatic indirect: unequal brace dose | Single-study estimate (no pooling) | 1 | Meng 2026 (FTS-021) | 2.240 (1.633 to 2.847) | NA | tau2=0.0000; I2=0.0% |
| Cobb exploratory imputed-SD stratum: Kyrkousis 2024 | Single-study estimate (no pooling) | 1 | Kyrkousis 2024 (FTS-019) | 2.460 (0.370 to 4.550) | NA | tau2=0.0000; I2=0.0% |
| Cobb exploratory abstract-limited imputed-SD stratum: Wu 2023 | Single-study estimate (no pooling) | 1 | Wu 2023 (FTS-015) | 3.120 (1.358 to 4.882) | NA | tau2=0.0000; I2=0.0% |
| Cobb exploratory imputed-SD sensitivity r=0.50 | Fixed-effect inverse-variance | 4 | Lee 2026 (FTS-008); Yao 2024 (FTS-016); Kyrkousis 2024 (FTS-019); Wu 2023 (FTS-015) | 3.520 (2.333 to 4.707) | NA | tau2=0.0000; I2=0.0% |
| Cobb exploratory imputed-SD sensitivity r=0.50 | DerSimonian-Laird random-effects | 4 | Lee 2026 (FTS-008); Yao 2024 (FTS-016); Kyrkousis 2024 (FTS-019); Wu 2023 (FTS-015) | 3.709 (2.141 to 5.276) | -0.180 to 7.598 | tau2=0.8543; I2=33.8% |
| Cobb exploratory imputed-SD sensitivity r=0.50 | REML random-effects | 4 | Lee 2026 (FTS-008); Yao 2024 (FTS-016); Kyrkousis 2024 (FTS-019); Wu 2023 (FTS-015) | 3.728 (2.114 to 5.343) | -0.375 to 7.832 | tau2=0.9845; I2=33.8% |
| Cobb exploratory imputed-SD sensitivity r=0.50 | DerSimonian-Laird Hartung-Knapp | 4 | Lee 2026 (FTS-008); Yao 2024 (FTS-016); Kyrkousis 2024 (FTS-019); Wu 2023 (FTS-015) | 3.709 (1.124 to 6.293) | -0.180 to 7.598 | tau2=0.8543; I2=33.8% |
| Cobb exploratory imputed-SD sensitivity r=0.50 | REML Hartung-Knapp | 4 | Lee 2026 (FTS-008); Yao 2024 (FTS-016); Kyrkousis 2024 (FTS-019); Wu 2023 (FTS-015) | 3.728 (1.107 to 6.350) | -0.375 to 7.832 | tau2=0.9845; I2=33.8% |
| Cobb exploratory imputed-SD sensitivity r=0.50 | Leave-one-out DL; omitted Lee 2026 (FTS-008) | 3 | Yao 2024 (FTS-016); Kyrkousis 2024 (FTS-019); Wu 2023 (FTS-015) | 3.013 (1.708 to 4.318) | 0.148 to 5.879 | tau2=0.0000; I2=0.0% |
| Cobb exploratory imputed-SD sensitivity r=0.50 | Leave-one-out DL; omitted Yao 2024 (FTS-016) | 3 | Lee 2026 (FTS-008); Kyrkousis 2024 (FTS-019); Wu 2023 (FTS-015) | 3.581 (1.816 to 5.347) | -2.505 to 9.668 | tau2=1.1896; I2=49.0% |
| Cobb exploratory imputed-SD sensitivity r=0.50 | Leave-one-out DL; omitted Kyrkousis 2024 (FTS-019) | 3 | Lee 2026 (FTS-008); Yao 2024 (FTS-016); Wu 2023 (FTS-015) | 4.364 (2.315 to 6.414) | -2.166 to 10.895 | tau2=1.2101; I2=35.0% |
| Cobb exploratory imputed-SD sensitivity r=0.50 | Leave-one-out DL; omitted Wu 2023 (FTS-015) | 3 | Lee 2026 (FTS-008); Yao 2024 (FTS-016); Kyrkousis 2024 (FTS-019) | 4.280 (1.685 to 6.874) | -4.783 to 13.342 | tau2=2.6842; I2=52.0% |
| ATR exploratory imputed-SD stratum: Kyrkousis 2024 | Single-study estimate (no pooling) | 1 | Kyrkousis 2024 (FTS-019) | 3.320 (2.369 to 4.271) | NA | tau2=0.0000; I2=0.0% |
| ATR exploratory abstract-limited imputed-SD stratum: Wu 2023 | Single-study estimate (no pooling) | 1 | Wu 2023 (FTS-015) | 1.120 (0.260 to 1.980) | NA | tau2=0.0000; I2=0.0% |
| ATR exploratory imputed-SD sensitivity r=0.50 | Fixed-effect inverse-variance | 3 | Lee 2026 (FTS-008); Kyrkousis 2024 (FTS-019); Wu 2023 (FTS-015) | 2.113 (1.523 to 2.703) | NA | tau2=0.0000; I2=0.0% |
| ATR exploratory imputed-SD sensitivity r=0.50 | DerSimonian-Laird random-effects | 3 | Lee 2026 (FTS-008); Kyrkousis 2024 (FTS-019); Wu 2023 (FTS-015) | 2.184 (0.703 to 3.665) | -3.830 to 8.198 | tau2=1.3823; I2=82.3% |
| ATR exploratory imputed-SD sensitivity r=0.50 | REML random-effects | 3 | Lee 2026 (FTS-008); Kyrkousis 2024 (FTS-019); Wu 2023 (FTS-015) | 2.182 (0.826 to 3.539) | -3.241 to 7.606 | tau2=1.1097; I2=82.3% |
| ATR exploratory imputed-SD sensitivity r=0.50 | DerSimonian-Laird Hartung-Knapp | 3 | Lee 2026 (FTS-008); Kyrkousis 2024 (FTS-019); Wu 2023 (FTS-015) | 2.184 (-1.068 to 5.435) | -3.830 to 8.198 | tau2=1.3823; I2=82.3% |
| ATR exploratory imputed-SD sensitivity r=0.50 | REML Hartung-Knapp | 3 | Lee 2026 (FTS-008); Kyrkousis 2024 (FTS-019); Wu 2023 (FTS-015) | 2.182 (-0.796 to 5.160) | -3.241 to 7.606 | tau2=1.1097; I2=82.3% |
| ATR exploratory imputed-SD sensitivity r=0.50 | Leave-one-out DL; omitted Lee 2026 (FTS-008) | 2 | Kyrkousis 2024 (FTS-019); Wu 2023 (FTS-015) | 2.210 (0.054 to 4.366) | -21.273 to 25.694 | tau2=2.2060; I2=91.2% |
| ATR exploratory imputed-SD sensitivity r=0.50 | Leave-one-out DL; omitted Kyrkousis 2024 (FTS-019) | 2 | Lee 2026 (FTS-008); Wu 2023 (FTS-015) | 1.411 (0.514 to 2.308) | -5.674 to 8.497 | tau2=0.1016; I2=19.9% |
| ATR exploratory imputed-SD sensitivity r=0.50 | Leave-one-out DL; omitted Wu 2023 (FTS-015) | 2 | Lee 2026 (FTS-008); Kyrkousis 2024 (FTS-019) | 2.888 (1.767 to 4.010) | -6.999 to 12.776 | tau2=0.2783; I2=39.3% |

Positive mean differences favour Schroth or Schroth-based exercises plus bracing. Prediction intervals are descriptive and especially unstable when based on two or three studies.
