# Supplementary material for: Schroth exercises added to bracing for adolescent idiopathic scoliosis: a systematic review and meta-analysis
Source: Front Sports Act Living. 2026 Sep 23;8:1919698. doi: 10.3389/fspor.2026.1919698 (PMC13642753; doi:10.3389/fspor.2026.1919698)
Supplement: Supplementary file 1 [file Datasheet1.zip › Frontiers_Sports_Active_Living_submission_ready_2026-06-25_Frontiers_guidelines_polished/supplementary_material/search_strategy.docx]

# Final search strategy and search log

Review: Schroth exercises added to bracing for adolescent idiopathic scoliosis.

The final search was conducted between 18 May 2026 and 23 May 2026. Searches used scoliosis/AIS terms, Schroth or scoliosis-specific exercise terms, and brace/orthosis terms where supported by the database interface. No language or date restrictions were applied at the search stage. Age and PICO eligibility were applied during screening.

The accompanying search log, source manifest, PRISMA-S checklist, and protocol-deviation summary report final reproducible sources, dates, strategies, record counts, archive files, raw-export custody, deduplication-count derivation, substitute evidence where full export was unavailable, and prespecified sources that could not be searched. The revised search archive includes custody manifests, a CENTRAL partial-capture note, Scopus/SinoMed access-attempt log, reproducible Scopus/SinoMed rerun strategies and export templates, a PRESS-style search self-audit checklist, and an editor-facing repository manifest; available raw exports and deduplication outputs are placed in the separate editor-facing repository deposit folder.

## Completed electronic sources

- PubMed (PubMed), searched 2026-05-18, records retrieved: 125.
- ClinicalTrials.gov (ClinicalTrials.gov), searched 2026-05-18, records retrieved: 83.
- Wanfang Data (Wanfang advanced search), searched 2026-05-19, records retrieved: 44.
- Wanfang Data (Wanfang advanced search), searched 2026-05-19, records retrieved: 55.
- Wanfang Data (Wanfang advanced search), searched 2026-05-19, records retrieved: 62.
- Wanfang Data (Wanfang advanced search), searched 2026-05-19, records retrieved: 62.
- Wanfang Data (Wanfang advanced search), searched 2026-05-19, records retrieved: 69.
- Wanfang Data (Wanfang advanced search), searched 2026-05-19, records retrieved: 74.
- CNKI (CNKI advanced search), searched 2026-05-19, records retrieved: 446.
- Embase (Embase), searched 2026-05-21, records retrieved: 381.
- Web of Science Core Collection (Web of Science), searched 2026-05-22, records retrieved: 88.
- CINAHL Complete (EBSCOhost), searched 2026-05-22, records retrieved: 42.
- VIP Database (中文期刊服务平台), searched 2026-05-22, records retrieved: 181.
- PEDro (PEDro), searched 2026-05-22, records retrieved: 70.
- Cochrane CENTRAL (Cochrane Library), searched 2026-05-23, records retrieved: 338 search hits across 11 searches; 38 visible metadata records captured/imported.
- WHO ICTRP (ICTRP Search Portal Advanced Search), searched 2026-05-23, records retrieved: 133 raw hits across 12 searches; 91 unique registry IDs captured.

## Core English strategy

Scoliosis/AIS concept:
("Scoliosis" OR scoliosis OR "adolescent idiopathic scoliosis" OR AIS)

Schroth/PSSE concept:
(Schroth OR "Schroth exercises" OR "Schroth method" OR "Schroth-based" OR "scoliosis-specific exercise" OR "scoliosis-specific exercises" OR "physiotherapeutic scoliosis-specific exercise" OR "physiotherapeutic scoliosis-specific exercises" OR PSSE)

Brace/orthosis concept:
(brace OR braces OR bracing OR orthosis OR orthoses OR "spinal orthosis" OR "spinal orthoses" OR TLSO OR "Boston brace" OR Cheneau OR "Cheneau" OR "Rigo-Cheneau" OR "Rigo Cheneau")

## PubMed / MEDLINE

("Scoliosis"[Mesh] OR scoliosis[tiab] OR "adolescent idiopathic scoliosis"[tiab] OR AIS[tiab])
AND
(Schroth[tiab] OR "Schroth exercises"[tiab] OR "Schroth method"[tiab] OR "Schroth-based"[tiab] OR "scoliosis-specific exercise"[tiab] OR "scoliosis-specific exercises"[tiab] OR "physiotherapeutic scoliosis-specific exercise"[tiab] OR "physiotherapeutic scoliosis-specific exercises"[tiab] OR PSSE[tiab])
AND
(brace[tiab] OR braces[tiab] OR bracing[tiab] OR orthosis[tiab] OR orthoses[tiab] OR "spinal orthosis"[tiab] OR "spinal orthoses"[tiab] OR TLSO[tiab] OR "Boston brace"[tiab] OR Cheneau[tiab] OR "Rigo-Cheneau"[tiab])

## Embase

('scoliosis'/exp OR scoliosis:ti,ab OR 'adolescent idiopathic scoliosis':ti,ab OR AIS:ti,ab)
AND
(Schroth:ti,ab OR 'Schroth exercise':ti,ab OR 'Schroth exercises':ti,ab OR 'Schroth method':ti,ab OR 'Schroth-based':ti,ab OR 'scoliosis-specific exercise':ti,ab OR 'scoliosis-specific exercises':ti,ab OR 'physiotherapeutic scoliosis-specific exercise':ti,ab OR 'physiotherapeutic scoliosis-specific exercises':ti,ab OR PSSE:ti,ab)

The Embase export used a deliberately broad scoliosis plus Schroth/PSSE strategy; brace/orthosis eligibility was applied during screening to avoid missing studies with incomplete abstract indexing.

## Web of Science / CINAHL / Cochrane CENTRAL

TS or title/abstract/keyword searches combined the same scoliosis/AIS, Schroth/PSSE, and brace/orthosis concepts listed above. CENTRAL was searched in the Trials tab. CENTRAL counts were recorded, but full bulk export was unavailable because institutional authentication to full CENTRAL content failed; visible metadata and search logs were archived, and this partial capture is treated as an operational search limitation.

## PEDro

PEDro uses limited Boolean functionality, so the following short searches were run and deduplicated: scoliosis schroth; scoliosis psse; scoliosis specific exercise; scoliosis brace schroth.

## Chinese database strategy

CNKI, Wanfang and VIP used combinations of:
(脊柱侧弯 OR 脊柱侧凸 OR 青少年特发性脊柱侧弯 OR 特发性脊柱侧弯 OR AIS)
AND
(施罗斯 OR 施罗特 OR Schroth OR 脊柱侧弯特异性运动 OR 脊柱侧凸特异性运动 OR PSSE)
AND, where the interface allowed:
(支具 OR 矫形器 OR 脊柱矫形器 OR 矫形支具)

Because some Chinese interfaces retrieve more completely with two-term fuzzy topic searches, broader searches combining scoliosis terms with Schroth terms were also run and screened against the brace comparator PICO.

## Trial registries

ClinicalTrials.gov and WHO ICTRP were searched using scoliosis condition terms combined with Schroth, PSSE, scoliosis-specific exercise, brace, bracing, orthosis, Cheneau, and Gensingen terms. Recruitment status, date, language, country, and phase filters were not applied.

## Prespecified sources not searched because access was unavailable

Scopus and SinoMed/CBM were not completed because no institutional or personal access was available during the final search window and the 2026-06-03 access recheck. No Scopus or SinoMed/CBM search is claimed. To mitigate this limitation, access attempts were logged, exact rerun strategies and export templates were archived, available English and Chinese database exports and deduplication outputs were deposited, CENTRAL partial capture was documented, and reference-list/forward-citation checks were used as supplementary discovery methods. A PRESS-style search self-audit checklist was prepared for transparent review, but no external PRESS endorsement is claimed.

## Repository and audit note

If requested by the editorial office, the authors can provide a read-only repository containing the available raw exports, final deduplication library, duplicate-removal logs, screening workbooks, search logs, and source custody manifests. Sources that could not be exported or accessed are identified in the manifest rather than treated as completed searches.
